# Comparative transcriptome analysis reveals molecular regulation of salt tolerance in two contrasting chickpea genotypes

**DOI:** 10.3389/fpls.2023.1191457

**Published:** 2023-05-30

**Authors:** Hammad Aziz Khan, Niharika Sharma, Kadambot H.M. Siddique, Timothy David Colmer, Tim Sutton, Ute Baumann

**Affiliations:** ^1^UWA School of Agriculture and Environment, The University of Western Australia, Perth, WA, Australia; ^2^The UWA Institute of Agriculture, The University of Western Australia, Perth, WA, Australia; ^3^NSW Department of Primary Industries, Orange Agricultural Institute, Orange, NSW, Australia; ^4^School of Agriculture, Food and Wine, University of Adelaide, Adelaide, SA, Australia; ^5^Department of Primary Industries and Regions, South Australian Research and Development Institute (SARDI), Adelaide, SA, Australia

**Keywords:** chickpea (*Cicer arietinum* L.), salt stress, RNA-sequencing, transcriptome analysis, salt tolerance, gene sequence variation.

## Abstract

Salinity is a major abiotic stress that causes substantial agricultural losses worldwide. Chickpea (*Cicer arietinum* L.) is an important legume crop but is salt-sensitive. Previous physiological and genetic studies revealed the contrasting response of two desi chickpea varieties, salt-sensitive Rupali and salt-tolerant Genesis836, to salt stress. To understand the complex molecular regulation of salt tolerance mechanisms in these two chickpea genotypes, we examined the leaf transcriptome repertoire of Rupali and Genesis836 in control and salt-stressed conditions. Using linear models, we identified categories of differentially expressed genes (DEGs) describing the genotypic differences: salt-responsive DEGs in Rupali (1,604) and Genesis836 (1,751) with 907 and 1,054 DEGs unique to Rupali and Genesis836, respectively, salt responsive DEGs (3,376), genotype-dependent DEGs (4,170), and genotype-dependent salt-responsive DEGs (122). Functional DEG annotation revealed that the salt treatment affected genes involved in ion transport, osmotic adjustment, photosynthesis, energy generation, stress and hormone signalling, and regulatory pathways. Our results showed that while Genesis836 and Rupali have similar primary salt response mechanisms (common salt-responsive DEGs), their contrasting salt response is attributed to the differential expression of genes primarily involved in ion transport and photosynthesis. Interestingly, variant calling between the two genotypes identified SNPs/InDels in 768 Genesis836 and 701 Rupali salt-responsive DEGs with 1,741 variants identified in Genesis836 and 1,449 variants identified in Rupali. In addition, the presence of premature stop codons was detected in 35 genes in Rupali. This study provides valuable insights into the molecular regulation underpinning the physiological basis of salt tolerance in two chickpea genotypes and offers potential candidate genes for the improvement of salt tolerance in chickpeas.

## Introduction

Chickpea (*Cicer arietinum* L.) is a nutritious legume crop, but salinity limits its production in areas where it is widely grown (Ryan, 1997; Flowers et al., 2010). Climate change will intensify soil salinity globally (Shokat and Grosskinsky, 2019); therefore, improved salinity tolerance is essential for sustained chickpea productivity in major salt-affected regions worldwide. Chickpea is a salt-sensitive species (Flowers et al., 2010); however, despite its narrow genetic diversity between genotypes, some variation for salt tolerance has been reported (Vadez et al., 2007; Turner et al., 2013; Varshney et al., 2021), which can be exploited for varietal improvement. Developing salt-tolerant crop varieties requires effective genetic variations, selection procedures and insights into salt tolerance mechanisms (Munns and Tester, 2008). The physiological and biochemical changes under salt stress are modulated by numerous genes and mediated *via* highly complex gene regulatory networks (Vadez et al., 2012). Therefore, identifying candidate genes involved in key physiological processes of salinity tolerance could help direct gene selection in chickpea breeding programs.

Several chickpea studies have investigated transcriptional changes of numerous genes to understand the regulatory mechanisms for tolerance to salt and other abiotic stresses like heat, drought, desiccation, and cold (Mantri et al., 2007; Varshney et al., 2009; Molina et al., 2011; Jain et al., 2013; Garg et al., 2015; Garg et al., 2016; Kaashyap et al., 2018; Kudapa et al., 2018; Kumar et al., 2021; Kaashyap et al., 2022). These studies have explored many chickpea genotypes and tissues under various stress conditions; however, the research was limited to one developmental stage or a single genotype until recently, when RNA-seq was used to examine salt stress tolerance in four genotypes (Kumar et al., 2021). Some studies identified the role of certain genes in salt stress tolerance and adaptation by exploring gene expression profiles using qPCR in tolerant and susceptible chickpea genotypes (Singh et al., 2018; Arefian et al., 2019). In chickpeas, salinity tolerance levels and mechanisms vary among different accessions and cultivars (Vadez et al., 2007; Turner et al., 2013; Sweetman et al., 2020) and the molecular basis of gene regulation for salinity tolerance has not been investigated in all genetic material.

Salt tolerance is a complex trait in plants, typically achieved through osmotic stress tolerance, Na^+^ and Cl^−^ exclusion, Na^+^ and Cl^−^ tissue tolerance, and maintenance of an adequate tissue K^+^/Na^+^ ratio (Munns and Tester, 2008; Kronzucker and Britto, 2011). Ion exclusion is the ability of salt-stressed plants to keep toxic ions (Na^+^ and/or Cl^−^) at relatively low concentrations in shoots by retaining them in roots, unloading them from the xylem stream, or storing them away from leaf photosynthetic tissues (Munns and Tester, 2008; Roy et al., 2014). Tissue tolerance is the ability of cells and tissues to function while accumulating high Na^+^ and Cl^−^, presumably by compartmentalising ions at the cellular and intracellular levels (Flowers et al., 2015; Munns et al., 2016). In addition, plants tolerate salt stress using a suite of mechanisms such as maintaining Na^+^/K^+^ homeostasis, growth rate, photosynthesis, cell wall integrity, stress signalling and regulatory pathways, cell redox homeostasis, hormone regulation, carbon partitioning, and translocation (van Zelm et al., 2020). Identifying salt tolerance mechanisms in chickpeas requires dissecting the main components in combination with the associated adaptive responses and then identifying the genic regulation of these mechanisms.

Two desi chickpea cultivars, Genesis836 and Rupali, show contrasting phenotypes when exposed to salt, leading to differences in photosynthesis, growth, and seed yield (Khan et al., 2015; Khan et al., 2016; Khan et al., 2017; Kotula et al., 2019; Atieno et al., 2021). However, their shoot ion (Na^+^ or Cl^−^) concentration did not differ (Khan et al., 2015; Kotula et al., 2015; Khan et al., 2016), suggesting that ion exclusion (at shoot or leaf level) does not explain the salt tolerance difference between the two genotypes and that it could be due to differences in their tissue tolerance to shoot high Na^+^ (Khan et al., 2015; Kotula et al., 2015; Khan et al., 2016). Moreover, another study associated salt tolerance in Genesis836 with higher photosynthetic rates and less structural damage to chloroplasts than Rupali, possibly through Na^+^ exclusion from the photosynthetically active mesophyll cells and compartmentalising Na^+^ into the non-photosynthetic epidermal cells (Kotula et al., 2019). Thus, the physiological basis of salt tolerance in chickpeas appears to be driven by a combination of Na^+^ exclusion and tissue tolerance to Na^+^ in different leaf tissues. However, the molecular mechanisms underlying salt tolerance in these genotypes remain unexplored.

This study used RNA-sequencing (RNA-seq) of leaf tissues of the two physiologically well-characterised genotypes grown in control and 60 mM NaCl conditions and a comprehensive transcriptome analysis to garner in-depth and unique information on transcriptional reprogramming, pathways, and regulatory networks associated with salt tolerance. In addition, sequence variants between Genesis836 and Rupali were examined for the presence/absence of functional variants. To our knowledge, this is the first study to explore the chickpea leaf transcriptome in two chickpea genotypes contrasting in response to salt stress.

## Materials and methods

### Plant growth, stress treatment, and tissue sampling

Two genotypes of desi chickpea (salt-tolerant Genesis836 and salt-sensitive Rupali) were selected based on their physiological and seed yield data from previous experiments (Khan et al., 2015; Kotula et al., 2015; Khan et al., 2016; Atieno et al., 2021). The study was performed in a glasshouse (Waite Campus, The University of Adelaide, Adelaide, SA, Australia) with natural irradiation and photoperiod (22 ± 3°C). Plants were grown in plastic pots (5 L) with a continuously aerated nutrient solution (Khan et al., 2015). Fourteen-day-old seedlings were subjected to salt stress (60 mM NaCl, added in four 15 mM increments 24 h apart). The nutrient solution in all pots was renewed weekly across the 40 days of plant growth, both before and after onset of salt treatment. The pH (~7.0) was adjusted twice weekly using potassium hydroxide (KOH); however, the degree of change in pH was similar among the genotypes. Pots were arranged in a completely randomised design, with six biological replicates of each genotype and treatment maintained under controlled conditions.

Tissues were sampled for RNA-seq and ion concentrations as soon as the first symptom of leaf damage appeared on older leaves of salt-treated plants (20-day-old plants or 6 days after treatment). The second youngest fully expanded leaf (2nd YFEL) was collected from six different plants/pots for each treatment condition. Leaf tissues were harvested for RNA-seq, quickly frozen in liquid nitrogen, and stored at −80°C until RNA extraction. A similar leaf (2nd YFEL) was harvested at the same time from another set of plants (grown in the same pot) to measure leaf ion concentrations (see below). The plants were grown for another 20 days (40-day-old plants) to observe the growth response, after which shoots and roots were separated and oven-dried at 65°C for 48 h to measure dry mass. The phenotypic data were analysed by two-way analysis of variance (ANOVA) using Genstat (VSN International Ltd. Hemel Hempstead, UK), with the means compared for significant differences using LSD (least significant difference) at the 5% significance level.

### Tissue ion analysis

Leaf samples (2nd YFEL oven-dried at 65°C for 48 h) were ground to a fine powder and sub-sampled for analysis of Na^+^, K^+^, and Cl^−^ following as previously described (Munns et al., 2010). Tissue samples were extracted in 0.5 M HNO_3_ by shaking for 48 h at room temperature. Diluted samples of the extracts were analysed for Na^+^ and K^+^ using a flame photometer (Flame Photometer Model 420, Sherwood Scientific Ltd., Cambridge, UK) and Cl^−^ with a chloridometer (Chloride Analyzer Model 926S, Sherwood Scientific Ltd., Cambridge, UK).

### RNA preparation and Illumina sequencing

Total RNA was extracted from the 2nd YFEL of 24 samples using the RNeasy Plant Mini Kit (Qiagen), according to the manufacturer’s instructions. The quantity and quality of the RNA were assessed using Nanodrop Spectrophotometer (NanoDrop Technologies, Wilmington, USA) and Agilent 2100 BioAnalyzer (Agilent Technologies Inc., Santa Clara, CA, USA). Stranded Illumina TruSeq libraries were prepared using high-quality RNA (RNA integrity number ≥ 8) and run on a HiSeq 2500 (Australian Genome Research Facility Ltd., Melbourne, Vic, Australia) to generate paired-end reads with a length of 100 base pairs (bp). All 24 RNA libraries (2 genotypes × 2 treatments × 6 biological replicates) were spread across four lanes (four technical replicates), totalling 96 RNA-seq datasets produced in FASTQ format. The data discussed in this publication have been deposited in NCBI SRA as PRJNA798198 (https://www.ncbi.nlm.nih.gov/sra/PRJNA798198).

### Read mapping

Raw data were subjected to quality control using FastQC version 0.11.2 (http://www.bioinformatics.babraham.ac.uk/projects/fastqc/) with the adapter sequences removed using Trimmomatic version 0.30 (Bolger et al., 2014). Trimming was performed in paired-end mode with min_len = 50, clip_seed_mm = 1, palindromeClipThreshold = 30, simpleClipThreshold = 10, and -phred33. Trimmed reads were mapped to the *C. arietinum* CDC Frontier *kabuli* version 2.6.3 reference genome (Edwards, 2016) using STAR version 2.7.3a (Dobin et al., 2013). The genome indices were generated with the reference genome and its corresponding annotation gff3 (general feature format) file with default parameters, except –sjdbOverhang 99, –sjdbGTFtagExonParentTranscript Parent, and –sjdbGTFtagExonParentGene ID using genomeGenerate mode. Next, mapping was performed with alignReads mode using the following parameters: –outSAMtype BAM SortedByCoordinate, –outSAMstrandField intronMotif, –outSAMprimaryFlag AllBestScore, –outFilterMultimapNmax 3, –outFilterMismatchNmax 2, –outFilterMatchNmin 70, –outFilterIntronMotifs RemoveNoncanonicalUnannotated, –alignIntronMin 30, –alignIntronMax 20000, –alignMatesGapMax 10000, –outSAMattrRGline ID, and –alignEndsType EndToEnd. The read alignment bam files generated after mapping were merged for all technical replicates and sorted and indexed using SAMTools version 1.8 (Li et al., 2009) (http://samtools.sourceforge.net/).

### Differential expression analysis

Read counting for the genes was performed and a count matrix was created using the *featureCounts()* function of the Rsubread package in R (Liao et al., 2019). A metadata file containing the complete information for bam files including genotype and treatment for all 24 samples was created. Four experimental groups were created according to the genotype and treatment conditions: Genesis_control, Genesis_treated, Rupali_control, and Rupali_treated. Next, the *DGEList()* function from the edgeR package was used to calculate the counts per million (CPM) for each experimental group and *calcNormFactors()* function was used to calculate normalisation factors (Nikolayeva and Robinson, 2014). Genes with no aligned reads in any sample were filtered out.

Counts of aligned reads were normalised to CPM and fitted with a linear model using *Limma (Linear Models for Microarray and RNA-Seq Data)* (Ritchie et al., 2015) to identify high- and low-expressed genes. Linear models allow one to assess differential expression in the context of multi-factor designed experiments, resulting in meaningful comparisons using interaction models. A design matrix was created with a group-mean parametrisation approach for multi-level comparison as explained in the manual (https://www.bioconductor.org/packages/devel/bioc/vignettes/limma/inst/doc/usersguide.pdf ). A contrast matrix was created to identify differentially expressed genes (DEGs) between control and treated samples in (a) Rupali and (b) Genesis836, and in response to a (c) genotype effect, (d) salt treatment effect, and (e) genotype and treatment interaction effect. False discovery rate (FDR) corrections of *p*-values were carried out using the Benjamini and Hochberg method (Benjamini and Hochberg, 1995). A gene was considered differentially expressed if it showed a corrected *p*-value ≤ 0.01, AveExpr ≥ 0, and B ≥ 1. AveExpr is the average log_2_-expression level for that gene across all the samples in the experiment and the *B*-statistic (lods or *B*) is the log-odds that the gene is differentially expressed. The lists of DEGs in all five comparisons were generated along with their respective log_2_FC (fold change) values.

The categories of DEGs are described below, which explain how the differential expression analysis was performed and constitutes the structure of how we present the results:

#### Salt-responsive DEGs in Rupali

The genes exhibiting significant differences in experimental groups of Rupali (Rupali_control vs. Rupali_treated)

#### Salt-responsive DEGs in Genesis836

The genes exhibiting significant differences in experimental groups of Genesis836 (Genesis836_control vs. Genesis836_treated)

#### Salt-responsive DEGs

The genes exhibiting significantly higher expression in one salt treatment compared to the other, independent of plant genotype (both control samples vs. both treated samples)

#### Genotype-dependent DEGs

The genes exhibiting significantly higher expression in one genotype compared to the other, independent of salt treatment (both Rupali samples vs. both Genesis836 samples)

#### Genotype-dependent salt-responsive DEGs

The genes with significant genotype and treatment interaction effect (the genes responding differently to salt treatment and among the two genotypes)

### Functional annotation

Chickpea gene sequences were retrieved from the reference sequence fasta file using the start and end coordinate information for genes given in the gff3 file. BLASTX searches of chickpea gene sequences were performed against *Arabidopsis thaliana* and *Medicago truncatula* Mt4.0v1 protein sequences with an e-value cutoff of 10^−15^. Protein sequences for *Arabidopsis* and *Medicago* were downloaded from https://www.arabidopsis.org/download_files/Proteins/TAIR10_protein_lists/TAIR10_pep_20101214 and https://phytozome.jgi.doe.gov/pz/portal.html#!info?alias=Org_Mtruncatula, respectively. BLASTX results were inspected for their top hit using an in-house perl script, and subsequently, putative annotations were added to the chickpea genes. Gene Ontology enrichment analysis and KEGG pathways were determined using the DAVID functional annotation tool (https://david.ncifcrf.gov/home.jsp ).

### Quantitative real-time PCR validation

qPCR was performed for eight genes (Ca30477, Ca13456, Ca02100, Ca14863, Ca10383, Ca29966, Ca19227, and Ca01215) randomly selected from RNA-seq data. RNA was extracted from leaf tissues and used for cDNA synthesis using SuperScriptIII reverse transcriptase (Invitrogen, Carlsbad, CA, USA). The gene-specific primers for qRT-PCR were designed using AlleleID software (Premier Biosoft International, Palo Alto, CA, USA). qPCR was performed as described previously (Ferdous et al., 2015), on all samples with three technical replicates for each of the six biological replicates. The transcript levels of each gene were normalised with the transcript levels of the three reference genes: elongation factor 1-alpha (*EF1α*; GenBank accession # AJ004960), heat shock protein 90 (*HSP90*; GenBank accession # GR406804), and glyceraldehyde-3-phosphate dehydrogenase (*GAPDH*; GenBank accession # AJ010224) (Garg et al., 2010).

### Identifying variants in two genotypes

The bam files generated by the STAR aligner were sorted by coordinates and indexed using SAMtools version 1.2. Next, bcftools mpileup and call was used to identify variants that were annotated with SnpEff (version 5.1). Only variants with quality ≥ 20, depth ≥ 10, and annotated as high and moderate categories were considered.

## Results

### Phenotypic and physiological responses to salt stress

In the control, Rupali had 25.2% and 16.6% higher shoot and root dry masses, respectively, than Genesis836 (**Table 1**). Salt stress severely reduced plant growth in both genotypes; however, Genesis836 had a higher shoot and root dry mass (34.3% and 45.8% of controls, respectively) than Rupali (8% and 10.8% of controls, respectively). Thus, the tolerant genotype showed better growth under salt stress after 26 days of treatment.

**Table 1:** Dry mass per plant of shoots and roots and ion concentrations in the 2nd youngest fully expanded leaf of two genotypes of chickpea (Rupali and Genesis836) grown in control and salt (60 mM NaCl) treatments for 26 days.

Treatment	Genotype	Dry mass per plant (g)		Ion concentration (μmol g^−1^ dry mass)			
		Shoots	Roots	Leaf Na^+^	Leaf Cl^−^	Leaf K^+^	Leaf K^+^/Na^+^
Control	Rupali	12.4 ± 1.5	2.8 ± 0.5	23 ± 005	65 ± 6	492 ± 32	23.8 ± 4.7
	Genesis836	9.9 ± 1.2	2.4 ± 0.2	14 ± 003	65 ± 6	506 ± 29	38.4 ± 6.4
60 mM NaCl	Rupali	1.0 ± 0.1	0.3 ± 0.1	604 ± 110	1,101 ± 182	480 ± 18	0.9 ± 0.1
	Genesis836	3.4 ± 0.1	1.1 ± 0.1	667 ± 073	1,153 ± 61	432 ± 41	0.7 ± 0.1
LSD (5%)							
Genotype		n.s.	n.s.	n.s.	n.s.	n.s.	5.8*
Treatment		1.5***	0.4***	98***	142***	n.s.	5.8***
Genotype × Treatment		2.1**	0.6**	n.s.	n.s.	n.s.	8.3*

Plants were grown in nutrient solution culture and treatments were imposed on 14-day-old plants. Leaves were sampled after 6 days of treatments whereas dry mass per plant of shoots and roots was measured after 25 days of treatments. Values are means ± SE (*n* = 6). The least significant differences (LSD) for genotype, treatment, and treatment × genotype interaction are given at the bottom of each column of data (*p* = 0.05). ***p < 0.001, **p < 0.01, *p < 0.05, n.s. = non-significant.

The salt treatment increased leaf Na^+^ and Cl^−^ concentrations (tissue dry mass basis) compared to controls, which did not differ between the two genotypes (**Table 1**). Moreover, the salt treatment did not affect leaf K^+^ concentration in either genotype. However, the salt treatment significantly decreased the leaf K^+^/Na^+^ ratio in both genotypes due to the increased leaf Na^+^ concentration (**Table 1**). Genesis836 had a higher leaf K^+^/Na^+^ ratio for control plants than Rupali, but both genotypes had similar ratios in the salt treatment.

### RNA-sequencing and identification of DEGs

RNA-seq of 96 leaf samples (2 genotypes × 2 treatments × 6 biological replicates × 4 technical replicates) generated more than 22 million paired-end 100-bp reads per sample. After trimming the reads to remove adapter contamination and low-quality bases, 95%−97% of high-quality reads were retained for each sample, with >87.3% uniquely aligned to the chickpea reference genome (CDC Frontier *kabuli* version 2.6.3) containing 33,351 genes (Edwards, 2016). Only 4.9% of reads were multi-mapped at various positions while the remaining 7.8% did not align with the reference genome. **Supplementary Table S1** summarises the generated sequence data, trimmed reads, and aligned reads.

**Figure 1:**
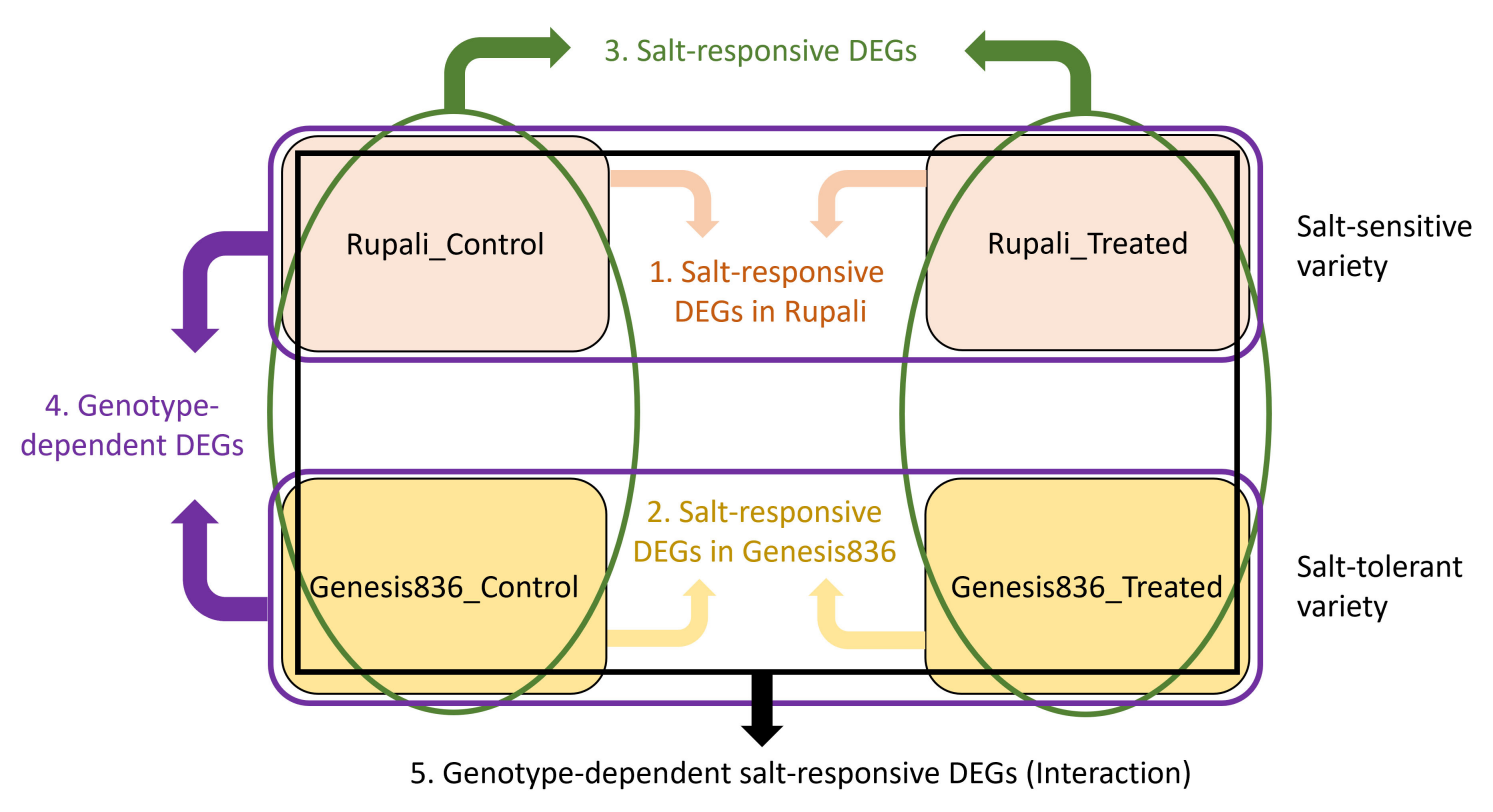
The four experimental groups (Rupali_Control, Rupali_treated, Genesis836_Control, and Genesis836_treated) were compared by LIMMA in five ways (shown as five colours) to test each contrast resulting in a list of DEGs. (1) Salt-responsive DEGs in Rupali = Rupali_Treated vs. Rupali_Control, (2) salt-responsive DEGs in Genesis836 = Genesis_Treated vs. Genesis_Control, (3) salt-responsive DEGs = (Rupali_Treated and Genesis_Treated) vs. (Rupali_Control and Genesis_Control), (4) genotype-depevdent DEGs = (Rupali_Treated and Rupali_Control) vs. (Genesis_Treated and Genesis_Control), and (5) genotype-dependent salt-responsive DEGs = (Rupali_Treated and Rupali_Control) vs. (Genesis_Treated and Genesis_Control).

Differential expression analysis was performed on four experimental groups (Genesis_control, Genesis_treated, Rupali_control, and Rupali_treated) using linear models resulting in effective and useful comparisons. **Figure 1** shows the comparisons done and resultant five categories of DEGs: (1) salt-responsive DEGs in Rupali, (2) salt-responsive DEGs in Genesis836, (3) salt-responsive DEGs, (4) genotype-dependent DEGs, and (5) genotype-dependent salt-responsive DEGs (interaction).

### Salt-responsive DEGs in Rupali and Genesis836

**Figure 2:**
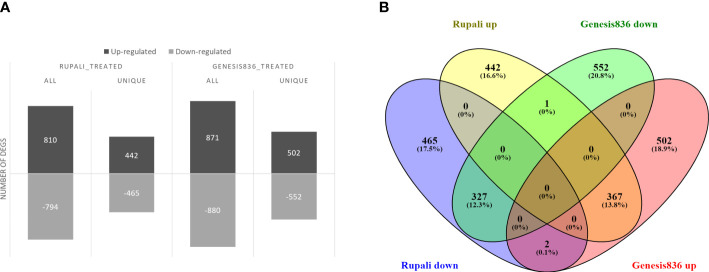
**(A)** Salt-responsive DEGs in two chickpea varieties, salt-sensitive Rupali and salt-tolerant Genesis836, and **(B)** common and unique salt-responsive DEGs in Rupali and Genesis836.

Salt stress significantly altered gene expression between control and treated samples in both genotypes. Rupali had 1,604 salt-responsive genes (**Supplementary Table S2**), of which 810 were upregulated and 794 were downregulated (**Figure 2A**). Similarly, salt stress resulted in 1,751 DEGs in Genesis836 (**Supplementary Table S3**) with 871 upregulated and 880 downregulated (**Figure 2A**). Rupali and Genesis836 had 697 common salt-responsive genes; Rupali had 907 and Genesis836 had 1,054 unique salt-responsive genes (**Figure 2B**).

Functional annotation and GO (gene ontology) enrichment analysis (**Supplementary Table S4**) revealed that the biological processes of Rupali’s downregulated genes were involved in the oxidation–reduction process, protein phosphorylation, and photosynthesis. In contrast, the highly represented GO terms for most of the upregulated DEGs were translation, response to salt stress, response to cytokinin, ribosome biogenesis, cell redox homeostasis, and leaf morphogenesis. For Genesis836, the enriched GO terms of downregulated genes included protein phosphorylation, protein ubiquitination, response to light, phosphorylation, response to ethylene, response to jasmonic acid, and photosynthesis. In contrast, most of the upregulated genes were associated with response to salt stress, response to heat, cell-redox homeostasis, response to high-intensity light, and transport.

Interestingly, the 697 common salt-responsive genes in the two genotypes (**Figure 2B**) had the same direction of expression change in both genotypes, suggesting a similar primary salt response mechanism in both the tolerant and susceptible genotypes, except for three transcription factor (TF) genes (Ca10694, Ca24486 and Ca07280) that exhibited the opposite expression patterns [i.e., genes induced in one genotype were repressed in the other (discussed later in the genotype-dependent salt-responsive genes section)]. Other common salt-responsive genes in the two chickpea genotypes included ABC transporter family proteins, alternative oxidase family proteins, vacuolar H^+^-pumping ATPase, CIPKs, chloride channel proteins, dehydration-responsive genes, electron transport family proteins, heat shock factors, cyclic nucleotide-gated cation channel proteins, and voltage-dependent anion channel proteins, with rRNA processing, ribosome biogenesis, embryo development ending in seed dormancy, and transport being the enriched GO categories associated with these groups of genes. This suggests that these common salt-responsive genes provide cellular membrane stability, signal transduction, stress response, and transporter roles and are associated with rendering key functions under salt stress (**Supplementary Table S4**).

**Figure 3:**
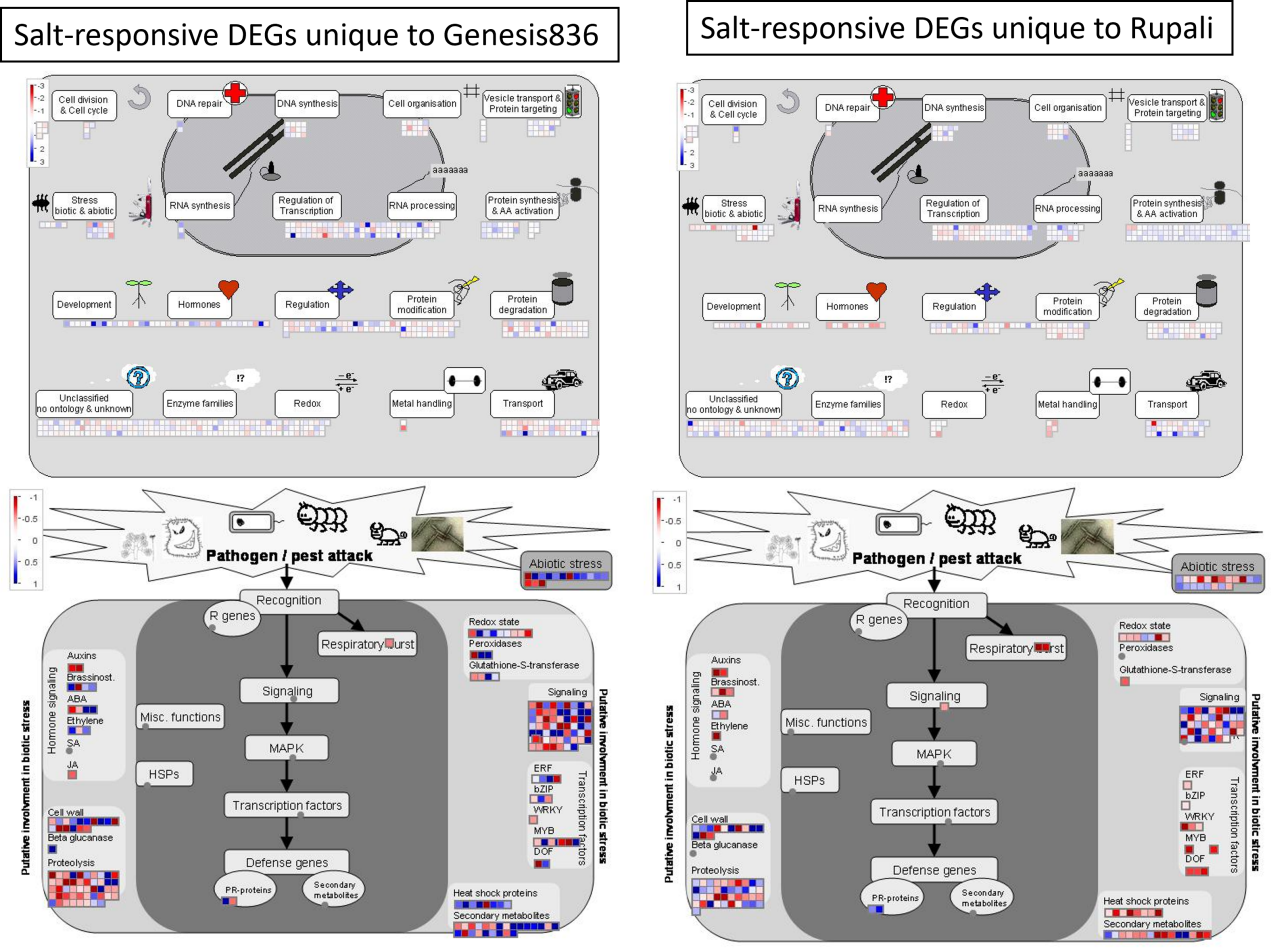
MapMan pathway views of salt-responsive genes unique to Genesis836 and Rupali.

In contrast, the salt-responsive genes unique to Rupali and Genesis836 had striking differences in expression pattern as delineated by MapMan pathway views (**Figure 3**). Upregulated salt-responsive DEGs unique to Genesis836 were mainly involved in stress (biotic and abiotic), development, hormones, RNA synthesis and processing, regulation, and redox. In contrast, salt-responsive DEGs unique to Rupali were downregulated in the same functional categories. Detailed pathway views showed that heat shock proteins, DEGs encoding TFs (ERF, bZIP, WRKY, MYB, and DOF) and secondary metabolites were mainly upregulated in Genesis836 and downregulated in Rupali. In Genesis836, unique downregulated DEGs were associated with proteolysis and signalling, while unique upregulated DEGs in Rupali were involved in protein synthesis and amino acid activation, vesicle transport and DNA synthesis. GO analysis revealed that unique salt-responsive genes in Rupali were associated with translation, embryo development ending in seed dormancy, response to cold, response to cytokinin, and mRNA processing. In contrast, the unique genes in Genesis836 were involved in the regulation of transcription, DNA-templated, response to salt stress, protein phosphorylation, metabolic process, response to light stimulus, response to cold, protein folding, flower development, circadian rhythm, and transmembrane transport (**Supplementary Table S4**).

### Salt-responsive DEGs

The salinity treatment affected the expression of 3,376 genes (**Supplementary Table S6**). Mapman analysis showed that the downregulated genes in this category were mainly involved in cell division, stress (heat, light), IAA, ethylene signalling, and calcium regulation. In contrast, the upregulated genes were implicated in cell cycle, stress (drought, salt), jasmonate, and ABA signalling. The GO enrichment analysis revealed that genes with treatment-dependent expression differences were mainly associated with response to light, glycolytic processes, stomatal movement, translation initiation, lignin biosynthesis, and chloroplast organisation (**Supplementary Table S4**). We did not explore this DEGs category further as investigating genes that represent common or general salt responses was not of interest.

### Genotype-dependent DEGs

**Figure 4:**
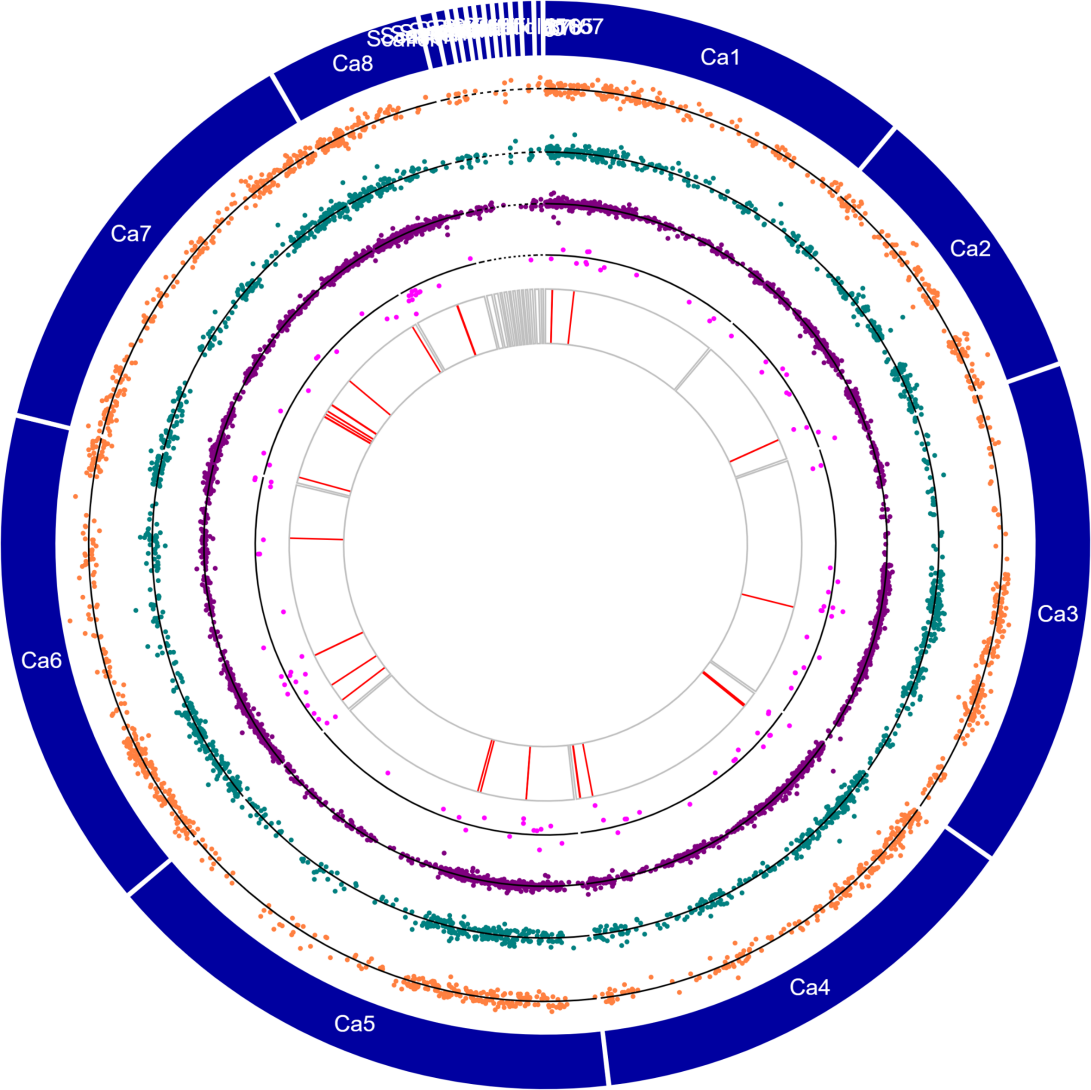
Circos plot showing chromosomes (Ca1–Ca8) and contigs (see genome description for details) in the outermost circle, orange dotplots represent the logFC values of salt-responsive genes unique to Rupali, green dotplots represent the logFC values of salt-responsive genes unique to Genesis836, purple dotplots represent the logFC values of DEGs with genotype-dependent expression, pink dotplots represent the logFC values of genotype-dependent salt-responsive genes, and red lines represent the genes with stop-gain mutations.

Comparative transcriptome analysis of the two chickpea genotypes revealed divergent gene expression of 4,170 DEGs, representing the genes expressed in both genotypes (**Figure 4**; **Supplementary Table S5**). Of these, 2,322 genes had higher expression in control and treated Genesis836 (↑Genesis836 expression) than the Rupali control and treated samples. Meanwhile, 1,848 genes had higher expression in Rupali (↑Rupali expression) than the Genesis836 samples. MapMan analysis of these DEGs indicated that a large fraction of genes with ↑Genesis836 expression were involved in DNA synthesis, RNA processing, protein synthesis and activation, and cell redox (thioredoxin), while most of the DEGs with ↑Rupali expression were light-responsive, receptor kinases, and involved in cell redox (glutaredoxin), cytokinin signalling, and calcium regulation. In this category, 767 DEGs exhibit at least a twofold change in expression with 401 and 366 genes highly expressed in Genesis836 and Rupali samples (control and treated), respectively.

#### DEGs contributing to the difference in salt response mechanism in two genotypes

GO and pathway analysis has highlighted some important functional categories for the unique Genesis836 and Rupali salt-responsive DEGs (**Table 2**) and DEGs exhibiting genotype-dependent expression (**Table 3**), which are discussed below. The categories below indicate the main differences between the genotypes in their response mechanism to salt stress.

**Table 2:** Salt-responsive DEGs unique to Rupali and Genesis836 contributing to their difference in salt response mechanism.

Functional Category	Salt-responsive DEGs unique to Rupali			Salt-responsive DEGs unique to Genesis836		
**Ion transport**	Gene ID	logFC	Annotation	Gene ID	logFC	Annotation
	Ca26987	0.96	Cyclic nucleotide-gated ion channel (CNGC2)	Ca04418	0.87	Chloride conductance regulatory protein Icln
	Ca25594	0.60	Vacuolar Ca2+/H+ exchanger (CAX1)	Ca33135	0.27	Vacuolar proton-transporting ATPase complex (VMA21-like)
	Ca21958	0.45	Cation-transporting ATPase	Ca11046	0.27	Na+/H+ exchanger 1 (NHX2)
	Ca18682	0.39	Vacuolar H+-translocating inorganic pyrophosphatase (AVP1)	Ca28033	−0.44	Cation efflux family protein (MTP11)
	Ca10473	0.33	Sodium/calcium exchanger (NCL)	Ca30428	−0.46	Cation-transporting ATPase
	Ca29678	−0.39	CBL-interacting protein kinase (SOS3-like CIPK11)	Ca26700	−0.47	Cation/H+ exchanger/antiporter (CHX3)
	Ca17275	−0.55	Cyclic nucleotide-gated ion channel (CNGC12)	Ca17299	−0.48	Cation-transporting ATPase
	Ca09655	−0.55	Cation efflux family protein	Ca00215	−0.87	Cation/H+ exchanger/antiporter (CHX18)
	Ca17327	−0.70	Cyclic nucleotide-gated ion channel (CNGC12)	Ca24768	−1.01	Proton-transporting ATPase
	Ca25729	−0.73	CBL-interacting protein kinase (SOS3-like CIPK11)			
	Ca01523	−0.76	High-affinity potassium transporter (HKT1)			
**Photosynthesis**	Ca07874	1.05	Plastidic ATP/ADP-transporter-like protein	Ca07255	0.54	Plastid transcriptionally active 12
	Ca11857	1.01	Chlorophyll A-B binding protein	Ca13535	0.37	Plastid division1 protein
	Ca11745	0.87	Plastidic pyruvate	Ca21550	0.30	Light-harvesting complex I chlorophyll A/B-binding protein
	Ca10820	0.67	Plastid developmental protein DAG	Ca21586	0.26	Plastid transcriptionally active 6
	Ca05526	0.52	Plastidic ATP/ADP-transporter-like protein	Ca31566	−0.30	Chloroplast curvature thylakoid 1B protein
	Ca31045	0.44	Rubisco methyltransferase family protein	Ca13018	−0.32	Plastid-lipid associated protein PAP
	Ca04693	0.43	Plastid transketolase	Ca31678	−0.33	High chlorophyll fluorescence phenotype 173
	Ca01477	0.40	Plastid specific ribosomal protein PSRP-3/Ycf65	Ca18504	−0.35	High chlorophyll fluorescent 107
	Ca29747	0.36	Cytosolic NADP+-dependent isocitrate	Ca06920	−0.36	Light harvesting complex photosystem II subunit 6
	Ca21261	0.32	Plastid ribosomal protein	Ca05670	−0.36	Photosynthetic NDH-dependent cyclic electron flow 1 protein
	Ca10728	0.26	Chloroplast stem-loop binding	Ca27911	−0.39	Chloroplast curvature thylakoid 1B protein
	Ca11065	−0.31	Thylakoid lumen 18.3 kDa protein	Ca01689	−0.40	Photosystem I light harvesting complex gene 1
	Ca31108	−0.32	Thylakoid membrane slr0575-like protein	Ca24996	−0.40	Plastid-lipid associated protein PAP
	Ca06895	−0.38	Plastid-lipid associated protein PAP	Ca08555	−0.40	Light-harvesting chlorophyll B-binding protein 3
	Ca18127	−0.39	Plastid phosphofructokinase family protein	Ca08914	−0.53	Photosystem I light harvesting complex gene 6
	Ca12317	−0.46	Photosystem II reaction center PsbP family protein	Ca13489	−0.86	Pheophorbide a oxygenase family protein with Rieske [2Fe-2S] domain
	Ca10587	−0.51	Photosystem II reaction center protein	Ca25182	−0.86	Chloroplast Ycf2; ATPase
	Ca14961	−0.57	Light-harvesting complex I chlorophyll A-B binding family protein	Ca30802	−0.90	Phosphoenolpyruvate (pep)/plastid phosphate translocator 2
	Ca03197	−0.60	Rubisco methyltransferase family protein			
	Ca06887	−0.61	Plastid-lipid associated protein PAP			
	Ca10288	−0.87	Photosystem I assembly protein Ycf3			
	Ca11095	−0.87	Photosystem II reaction center protein PsbP			
	Ca25170	−1.02	Photosystem II reaction center protein			
	Ca22712	−1.09	Photosystem II reaction center protein			
**Osmotic and dehydration-responsive genes**	Ca07247	0.61	Early-responsive to dehydration (ERD) protein	Ca03142	1.45	Late embryogenesis abundant (LEA) protein
	Ca18937	0.54	Delta-1-pyrroline-5-carboxylate dehydrogenase (P5CDH)	Ca24138	1.44	Polyamine oxidase 1 (PAO1)
	Ca23258	−0.34	Proline synthetase associated protein	Ca20393	1.22	Dehydration-responsive RD22-like protein
	Ca04683	−0.48	Arginase family protein (ARGAH1)	Ca03667	0.75	Myo-inositol-1-phosphate synthase 2 (MIPS2)
	Ca30779	−0.83	Raffinose synthase family protein (SIP1)	Ca21682	0.70	Spermidine synthase 3, polyamine biosynthetic process (SPDS3)
	Ca26838	−0.88	Proline dehydrogenase	Ca03022	0.64	Early-responsive to dehydration stress protein (ERD4)
	Ca26841	−0.94	Proline dehydrogenase	Ca01417	0.63	Proline extensin-like receptor kinase 1 (PERK1)
				Ca23212	0.61	Dehydration-induced protein (ERD15)
				Ca19250	0.60	Raffinose synthase family protein (SIP1)
				Ca27897	0.57	Delta-1-pyrroline-5-carboxylate synthetase 2
				Ca06701	0.55	Glutamine synthetase 1;4 (GLN1;4)
				Ca11285	0.48	Late embryogenesis abundant protein (LEA) family protein
				Ca17337	0.46	Late embryogenesis abundant protein (LEA) family protein
				Ca23033	0.27	Late embryogenesis abundant protein (LEA) family protein
				Ca00264	0.21	Dehydration-responsive protein
				Ca10462	−0.26	Glutamine amidotransferase-like superfamily protein
				Ca04970	−0.59	Trehalose-phosphate phosphatase
				Ca14629	−1.18	S-adenosyl-L-methionine-dependent methyltransferases protein (ACL5)
**Stress signalling and regulatory pathways**	Ca24711	0.31	Calcium-dependent protein kinase (CPK13)	Ca06774	2.24	Highly ABA-induced PP2C gene 2 (HAI2)
	Ca31905	−0.25	Calcineurin B-like protein (CBL2)	Ca20945	1.18	Abscisic acid responsive elements-binding factor 2 (ABF2/AREB1)
	Ca03519	−0.33	Calmodulin-domain kinase CDPK protein (CDPK2)	Ca06872	0.76	SNF1-related protein kinase 2.1 (SNRK2.1)
	Ca16701	−0.36	Calcineurin B-like protein (CBL10)	Ca13545	0.66	Regulatory components of ABA receptor 3 (RCAR3/PYL8)
	Ca00545	−0.36	Abscisic acid-activated signaling pathway (CEN1)	Ca02503	0.51	Abscisic acid responsive elements-binding factor 2 (ABF2/AREB1)
				Ca28788	0.31	Short root in salt medium 1 (RSA1)
	Ca30107	−0.58	ABA-responsive element (AREB3)	Ca17277	−0.30	CBL-interacting protein kinase 9, similar to SOS2 (CIPK9)
	Ca03741	−0.73	Serine/threonine-kinase SAPK1-like protein (SNRK2.4)	Ca15142	−0.44	Zeaxanthin epoxidase (ZEP) (ABA1)
	Ca13161	−0.81	CBL-interacting protein kinase (CIPK4, SnRK3.3)	Ca07390	−0.78	Mitogen-activated protein kinase 20 (MPK20)
				Ca14908	−0.82	Abscisic acid-responsive family protein (TB2/DP1, HVA22)
				Ca01048	−1.21	Abscisic acid receptor PYR1-like protein (PYR1/RCAR12)
**Cell redox homeostasis**	Ca01231	0.73	Glutathione S-transferase family protein	Ca12445	1.89	Glutathione S-transferase family protein (GSTL3)
	Ca15932	0.66	Nucleobase-ascorbate transporter	Ca20449	1.22	Glutathione S-transferase family protein (GSTL3)
	Ca26704	0.63	Glutathione S-transferase family protein	Ca21118	1.10	Glutaredoxin family protein (GRX)
	Ca15459	−0.33	Glutathione S-transferase (GSTZ2)	Ca05284	0.29	Glutaredoxin family protein (GRX)
	Ca04671	−0.39	Fe superoxide dismutase 3 (FSD3)	Ca03490	0.29	Glutathione S-transferase THETA 1 (GSTT1)
	Ca10585	−0.46	Glutathione dehydrogenase	Ca03004	0.25	Ascorbate peroxidase 3 (APX3)
	Ca31913	−0.65	Glutathione S-transferase (GSTZ2)	Ca06563	−0.52	Glutathione S-transferase (GSTU7)
	Ca17240	−0.88	Glutathione S-transferase	Ca31453	−0.55	Glutathione S-transferase family protein (GST30)
	Ca07284	−1.49	Glutaredoxin family protein (GRX)	Ca24778	−0.83	Glutaredoxin family protein (GRX)

**Table 3:** DEGs with genotype-dependent expression differences among Rupali and Genesis836 contributing to their difference in salt response mechanisms.

		DEGs with genotype-dependent expression differences			
Functional category		↑Rupali		↑Genesis836	
		Gene ID	Annotation	Gene ID	Annotation
**Ion transport**	**Sodium**	Ca29678	SOS3-interacting (CIPK11)	Ca14663	Calcineurin B-like protein (CBL10)
		Ca20032	SOS3-interacting (CIPK10)	Ca29649	Cation/H+ exchanger (CHX15)
		Ca13688	Plasma membrane H+-ATPase (HA1)	Ca10473	Sodium/calcium exchanger family protein
		Ca13694	Plasma membrane H+-ATPase (HA1)	Ca18734	CBL-interacting kinase (SOS2/CIPK24)
		Ca01523	High-affinity K+ transporter (HKT1)	Ca26023	Vacuolar-type H+-ATPase subunit B (VAB1)
		Ca21700	Calcineurin B-like protein (SOS3)	Ca33135	Vacuolar ATPase assembly protein (VMA21-like)
		Ca25729	SOS3-interacting (CIPK11)	Ca06383	Vacuolar proton ATPase A2 (VHA-A2)
		Ca20034	SOS3-interacting (CIPK11)	Ca12055	Na+/H+ antiporter (NHD1)
		Ca29676	SOS3-interacting (CIPK10)		
	**CNGCs**	Ca32080	Cyclic nucleotide gated channel (CNGC20)	Ca18209	Cyclic nucleotide gated channel (CNGC9)
		Ca17327	Cyclic nucleotide gated channel (CNGC12)		
		Ca17275	Cyclic nucleotide gated channel (CNGC12)		
	**Chloride**	Ca01155	Chloride channel C (CLC-C)	Ca04418	Chloride conductance regulatory protein ICln protein
				Ca05659	Chloride channel C (CLC-C)
				Ca24944	Chloride channel B (CLC-B)
	**Potassium**	Ca01696	K+ uptake transporter (KUP3)	Ca30848	Potassium transporter family protein
		Ca02200	K+ efflux antiporter 3 (KEA3)	Ca14669	K+ efflux antiporter 6 (KEA6)
		Ca30365	Outward-rectifying potassium channel (KCO1)	Ca02163	K+ uptake permease 7 (KUP7)
		Ca15849	Outward-rectifying potassium channel (KCO1)	Ca12821	Voltage-gated potassium channel subunit beta
**Photosynthesis**		Ca29856	Rubisco carboxylase/oxygenase activase (RCA)	Ca09247	Light-harvesting complex I chlorophyll A/B-binding protein (LHCB4.3)
		Ca01096	Light-harvesting complex I chlorophyll A/B-binding protein (LHCB5)	Ca05575	Post-illumination chlorophyll fluorescence (PIF1)
		Ca05313	Light-harvesting complex I chlorophyll A/B-binding protein (LHCB4.2)	Ca07502	Plastid lipid-associated protein (FIB)
		Ca13535	Plastid division 1 (PDV1)	Ca31619	Plastid lipid-associated protein (FIB)
		Ca00308	Thylakoid lumenal protein	Ca06875	Plastid developmental protein DAG
		Ca05670	NDH-dependent cyclic electron flow protein (NDH)	Ca31045	Rubisco methyltransferase family protein
		Ca17260	Light-harvesting complex I chlorophyll A/B-binding protein (LHCA2)	Ca27399	Sucrose synthase 3 (SUS3)
		Ca30421	Light-harvesting complex I chlorophyll A/B-binding protein (LHCB2.3)	Ca10460	Plastid developmental protein DAG
		Ca12317	Photosystem II reaction center PsbP family protein (PPL2)	Ca05526	Plastidic ATP/ADP-transporter-like protein
		Ca21063	Thylakoid lumenal 17.9 kDa protein	Ca10972	Rubisco accumulation factor 1
		Ca08555	Light-harvesting complex I chlorophyll A/B-binding protein (LHCB3)	Ca03918	RuBisCO large subunit-binding protein subunit alpha
		Ca18968	Plastid transcriptionally active (PTAC2)	Ca09821	RuBisCO large subunit-binding protein subunit beta
		Ca18416	Sucrose synthase 4 (SUS4)	Ca08711	Plastid transcriptionally active 14 protein
		Ca26111	Plastid movement impaired-like protein (PMI2)	Ca10830	Plastid-lipid associated protein (FIB4)
		Ca03595	Plastid-lipid-associated protein 7	Ca08571	Protochlorophyllide oxidoreductase (PORB)
		Ca17461	Curvature thylakoid 1B protein	Ca13080	Differentiation and greening-like protein (DAL1)
				Ca05298	Thylakoid ADP -ATP carrier protein
				Ca13380	Pyrophosphate-fructose-6-phosphate 1-Phosphotransferase
				Ca07255	Plastid transcriptionally active protein (PTAC12)
				Ca12430	Photosynthetic NDH subunit of lumenal
				Ca07361	Plastid developmental protein DAG
				Ca25742	Non-green plastid inner envelope membrane protein
				Ca01002	Plastid-lipid associated protein (PAP)
				Ca21550	Chlorophyll A-B binding family (NPQ4)
				Ca21586	Plastid transcriptionally active (PTAC6)
				Ca00066	Photosystem I subunit D-2 (PSAD-2)
**Osmotic and dehydration-responsive genes**		Ca19236	Drought-induced protein	Ca26119	Class I glutamine amidotransferase
		Ca04970	Trehalose-phosphate phosphatase (TPPG)	Ca03449	GMP synthase (glutamine-hydrolyzing)
		Ca03022	Early-responsive to dehydration stress (ERD) family protein	Ca24138	Polyamine oxidase (PAO1)
		Ca23618	Polyamine oxidase 2 (PAO2)	Ca29983	Early response-like dehydration-protein
		Ca26841	Proline dehydrogenase	Ca23212	Early response-like dehydration-protein
		Ca02177	Class I glutamine amidotransferase-like	Ca10386	Trehalose-6-phosphate synthase
		Ca20373	Dehydrin ERD14-like	Ca20393	Dehydration-responsive RD22-like protein
		Ca03667	Myo-inositol 1-phosphate synthase (MIPS2)	Ca29910	Proline transporter 1 (PROT1)
		Ca25782	Late embryogenesis abundant (LEA) protein	Ca06737	Polyamine oxidase 4 (PAO4)
		Ca06701	Glutamine synthetase 1;4 (GLN1;4)	Ca19219	Proline transporter 2 (PROT2)
		Ca14703	Early-responsive to dehydration stress (ERD) family protein	Ca12117	Trehalose phosphatase/synthase
		Ca16207	Trehalose-6-phosphate synthase	Ca19097	Myo-inositol polyphosphate 5-phosphatase
		Ca13645	Dehydration-responsive protein RD22	Ca03067	Proline transporter 1 (PROT1)
		Ca05155	Late embryogenesis abundant (LEA) protein	Ca06790	Delta1-pyrroline-5-carboxylate (P5CS1)
		Ca05154	Late embryogenesis abundant (LEA) protein	Ca20124	Class I glutamine amidotransferase-like
		Ca27912	Late embryogenesis abundant (LEA) protein	Ca30945	Early-responsive to dehydration stress (ERD) family protein
		Ca11326	Late embryogenesis abundant (LEA) protein	Ca26362	Pyrroline-5-carboxylate reductase (P5CR)
		Ca04033	Late embryogenesis abundant (LEA) protein	Ca12135	Glutamine-tRNA ligase, putative
		Ca21540	Myo-inositol 1-phosphate synthase (MIPS1)		
		Ca27389	Late embryogenesis abundant (LEA) protein		
		Ca20394	Dehydration-responsive RD22-like protein		
		Ca24842	Late embryogenesis abundant (LEA) protein		
**Stress signalling and regulatory pathways**		Ca30481	Calcium-dependent protein kinase (CPK6)	Ca14908	Abscisic acid-responsive (TB2/DP1)
		Ca26329	Calcium-dependent protein kinase (CRK1)	Ca13545	Abscisic acid receptor PYL9-like protein (PYL9)
		Ca18949	Calcium-dependent protein kinase (CPK30)	Ca02448	MAP kinase (MAPK6)
		Ca26415	Calcium-dependent protein kinase (CPK8)	Ca00557	CDPK-related kinase (CRK)
		Ca30122	CBL-interacting protein kinase (CIPK3)	Ca02503	Abscisic acid responsive factor (ABF2)
		Ca27310	Phosphatidic acid phosphatase (PAP2)	Ca24711	Calcium-dependent protein kinase (CPK13)
		Ca07182	CBL-interacting protein kinase (CIPK12)	Ca18734	CBL-interacting kinase (SOS2)
		Ca23968	Abscisic acid biosynthesis (ABA2)		
		Ca07803	CBL-interacting protein kinase (CIPK25)		
		Ca05580	Phosphatidic acid phosphatase (PAP2)		
		Ca19660	Calcium-dependent protein kinase (CPK33)		
		Ca17277	CBL-interacting protein kinase (CIPK9)		
		Ca01048	Abscisic acid receptor PYR1-like protein		
		Ca00527	Abscisic acid biosynthesis protein (ABA1)		
		Ca02486	CDPK-related kinase (CRK)		
		Ca03776	Mitogen-activated protein kinase (MAPK3)		
		Ca20975	Mitogen-activated protein kinase (MAPKKK5)		
**Cell redox homeostasis**		Ca03004	Ascorbate peroxidase 3 (APX3)	Ca14356	Glutathione S-transferase (GSTU1)
		Ca15459	Glutathione S-transferase (GSTU2)	Ca05730	Glutathione S-transferase (GSTU1)
		Ca11792	Transmembrane ascorbate ferrireductase	Ca01231	Glutathione S-transferase (GSTU1)
		Ca31963	Microsomal glutathione s-transferase	Ca10675	Glutathione reductase (GR)
		Ca06563	Glutathione S-transferase (GSTU7)	Ca18438	Glutathione S-transferase (GSTU24)
		Ca15694	Fe superoxide dismutase 2 (FSD2)		
		Ca03496	Glutathione peroxidase (GPX8)		
		Ca10743	Glutathione peroxidase (GPX5)		

↑Rupali indicate higher expression in Rupali compared to Genesis836 and ↑Genesis836 indicate higher expression in Genesis836 compared to Rupali.

#### Ion transport

**Figure 5:**
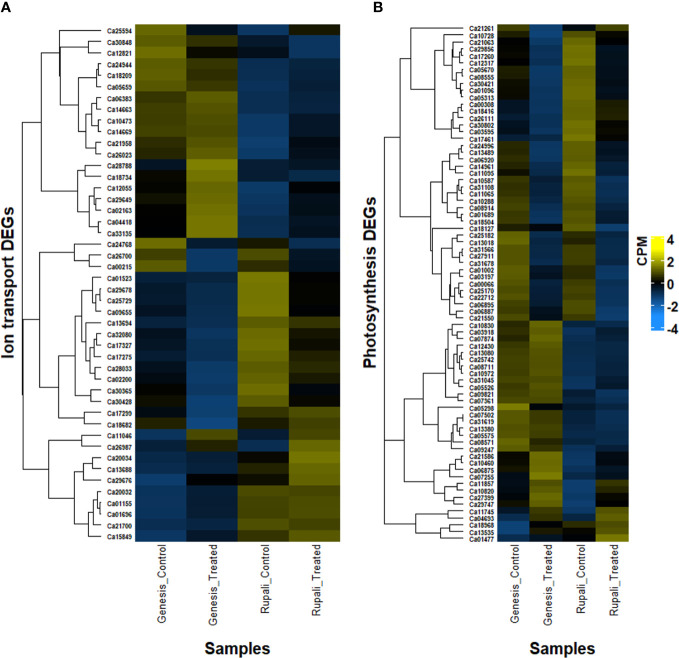
Heatmap of differentially expressed genes in two contrasting chickpea varieties, salt-sensitive Rupali and salt-tolerant Genesis836 involved in **(A)** ion transport and **(B)** photosynthesis.

Ionic stress is a critical component of salinity resulting from changes in sodium, potassium, and chloride homeostasis (Assaha et al., 2017); therefore, genes with a known role in ion (Na^+^, K^+^, and Cl^−^) transport, directly or indirectly, play a crucial role in determining salt tolerance in plants. **Figure 5A** shows the expression pattern of DEGs involved in ion transport, including 11 unique salt-responsive genes in Rupali and 17 genes with ↑Rupali expression, and 10 unique salt-responsive genes in Genesis836 and 16 genes with ↑Genesis836 expression (**Tables 2**, **3**). Salt-sensitive Rupali repressed an important gene involved in sodium exclusion, high-affinity potassium transporter (*HKT1* Ca01523) between control and treated samples, but maintained ↑Rupali expression relative to Genesis836. Similarly, the salt treatment downregulated two other genes in Rupali involved in sodium transport *via* the SOS pathway, encoding CBL-interacting protein kinases (*SOS3-like CIPK11*—Ca25729 and Ca29678), but exhibited ↑Rupali expression along with *SOS3* (Ca21700), *SOS3-interacting CIPK10* (Ca20032 and Ca29676) and *SOS3-interacting protein CIPK11* (Ca20034). In contrast, the salt treatment upregulated a gene encoding vacuolar H^+^-translocating inorganic pyrophosphatase (*AVP1*—Ca18682) in Rupali. Additionally, plasma membrane H^+^-ATPase (*AHA1*—Ca13688 and Ca13694) involved in sodium regulation across the plasma membrane exhibited genotype-dependent ↑Rupali expression. Other interesting DEGs from Rupali salt-responsive genes were potentially involved in cation transport, including sodium/calcium exchanger (*NCL*—Ca10473) and vacuolar Ca^2+^/H^+^ exchanger (*CAX1*—Ca25594).

Contrastingly, salt-tolerant Genesis836 upregulated two important genes involved in sodium transport, Na^+^/H^+^ exchanger 1 (*NHX2*—Ca11046) and vacuolar proton-transporting ATPase complex (*VMA21-like*—Ca33135). Genotype-dependent ↑Genesis836 expression was exhibited by sodium/calcium exchanger family protein (Ca10473), Na^+^/H^+^ antiporter (*NHD1*—Ca12055), vacuolar-type H^+^-ATPase (Ca26023 and Ca33135), and vacuolar proton ATPase A2 (*VHA-A2*—Ca06383). The salt treatment upregulated the short root in salt medium 1 (*RSA1*—Ca28788) in Genesis836. Downregulated unique DEGs from Genesis836 salt-responsive genes included proton-transporting ATPase (Ca24768), cation-transporting ATPase (Ca30428 and Ca17299—both delineated ↑Rupali expression), cation/H^+^ exchanger/antiporter (*CHX3/CHX18*—Ca26700 and Ca00215), and cation efflux family protein (*MTP11*—Ca28033 ↑Rupali expression), which are crucial for ion transport and maintaining ion homeostasis.

Chloride influx, transport, and regulatory mechanisms are other important aspects of plant salt tolerance studies. The salt treatment upregulated a gene encoding chloride conductance regulatory protein (*ICln*—Ca04418) in Genesis836, which also presented ↑Genesis836 expression. Similarly, chloride channel C (*ClC1*—Ca05659) and chloride channel B (*ClC*—Ca24944) also delineated ↑Genesis836 expression, while only one chloride channel C (*ClC1*—Ca01155) exhibited ↑Rupali expression.

Cyclic nucleotide-gated ion channel (CNGC) is an interesting category of proteins potentially involved in sodium transport. Interestingly, two CNGCs (Ca17327 and Ca17275) were downregulated in Rupali under salt stress but showed ↑Rupali expression along with Ca32080. One CNGC (Ca26987) was upregulated in control Rupali, while the other CNGC (Ca18209) exhibited ↑Genesis836 expression. Other ion transport DEGs with ↑Genesis836 expression included a sodium/calcium exchanger (*CHX*—Ca29649). Ca21958 and Ca09655 were among the unique salt-responsive DEGs in Rupali.

Regulation of K^+^ transport plays an important role in improving salinity stress tolerance in plants. Of the eight genes involved in K^+^ transport with genotype-dependent expression, four showed ↑Genesis836 [K^+^ transporter family protein (Ca30848), K^+^ efflux antiporter 6 (*KEA6*—Ca14669), K^+^ uptake permease 7 (*KUP7*—Ca02163), and voltage-gated K^+^ channel subunit beta (Ca12821)] and four showed ↑Rupali expression [K^+^ uptake transporter (*KUP3*—Ca01696), K^+^ efflux antiporter 3 (*KEA3*—Ca02200), and two outward-rectifying potassium channels (*KCO1*—Ca30365 and Ca15849)].

#### Photosynthesis

In Rupali, 24 genes related to photosynthetic activity were differentially regulated by salt stress and 16 photosynthesis-related genes exhibited genotype-dependent ↑Rupali expression (**Tables 2**, **3**; **Figure 5B**). In support of the observed growth reduction in Rupali, most DEGs associated with the Rubisco/thylakoid lumen, photosystem I and II assembly/reaction centre (Ca22712, Ca25170, Ca11095, Ca10288, Ca14961, Ca10587, Ca12317, Ca03197, Ca11065, and Ca31108), and plastid-lipid associated proteins (Ca06887, Ca18127, and Ca06895) were downregulated. Upregulated Rupali salt-responsive genes were mainly related to plastid development (Ca10820), plastidic ATP/ADP transport (Ca07874, Ca05526, and Ca29747), plastid pyruvate (Ca11745), plastid transketolase (Ca04693), plastid ribosomal proteins (Ca01477 and Ca21261), and chloroplast stem binding protein (Ca10728). The salt treatment showed ↑Rupali expression of light-harvesting complex I chlorophyll A/B-binding proteins LHCA2 (Ca17260), LHCB2.3 (Ca08555), LHCB3 (Ca30421), LHCB4.2 (Ca01096), and LHCB5 (Ca05313); however, salt stress repressed LHCB2.3 (Ca08555) in Genesis836.

Genesis836 had 18 unique salt-responsive DEGs related to photosynthesis (4 upregulated and 14 downregulated genes) and 26 genes with genotype-dependent ↑Genesis836 expression (**Tables 2**, **3**; **Figure 5B**). The salt-induced genes included a light-harvesting complex I chlorophyll A/B-binding protein (*CP22*—Ca21550) and three plastid development proteins (Ca07255, Ca13535, and Ca21586). Another gene LHCB4.3 (Ca09247) had 8.7-fold higher gene expression in Genesis836 than Rupali and is among the top 10 genes with ↑Genesis836 expression. Genesis836 also exhibited higher expression of plastid growth-related genes encoding plastid-lipid associated protein (*FIB/PAP*—Ca07502, Ca31619, Ca10830, and Ca01002), plastid transcriptionally active (*PTAC6*—Ca21586, *PTAC12* Ca07255, and *PTAC14* Ca08711), plastid developmental protein DAG (Ca06875, Ca07361, and Ca10460), and differentiation and greening-like protein (*DAL1*—Ca13080). Other notable photosystem-related genes with ↑Genesis836 expression included post-illumination chlorophyll fluorescence (*PIF1*—Ca05575), sucrose synthase 3 (*SUS3*—Ca27399), RuBisCO large subunit-binding protein subunit alpha and beta (Ca03918 and Ca09821), protochlorophyllide oxidoreductase (*PORB*—Ca08571), photosynthetic NDH subunit of luminal (Ca12430), chlorophyll A-B binding family (*NPQ4*—Ca21550), and photosystem I subunit D-2 (*PSAD-2*—Ca00066).

The downregulated unique salt-responsive genes in Genesis836 encoded for photosystem I and II light-harvesting complex (Ca06920, Ca01689, and Ca08914), high chlorophyll fluorescence phenotype (Ca31678 and Ca18504), chloroplast curvature thylakoid protein (Ca27911 and Ca31566), chloroplast Ycf2 ATPase (Ca25182), plastid-lipid proteins (Ca13018 and Ca24996), oxygenase family protein (Ca13489), and phosphoenolpyruvate (pep)/plastid phosphate translocator 2 (Ca30802). ↑Rupali expression was exhibited by genes encoding plastid division 1 (*PDV1*—Ca13535), thylakoid lumenal protein (Ca00308 and Ca21063), NDH-dependent cyclic electron flow protein (NDH Ca05670), photosystem II reaction centre PsbP family protein (*PPL2*—Ca12317), sucrose synthase 4 (*SUS4*—Ca18416), plastid movement impaired-like protein (*PMI2*—Ca26111), plastid-lipid-associated protein (Ca03595), and curvature thylakoid 1B protein (Ca17461). Notably, a different set of genes related to plastid development, photosystem, and photosynthesis process exhibited higher expression in both genotypes, indicating differences in photosynthetic processes to salt stress among the two genotypes.

#### Osmotic and dehydration-responsive genes

Another major functional category includes DEGs involved in osmotic stress tolerance (including genes involved in synthesising and transporting compatible solutes) and response to water deprivation. There were 18 unique salt-responsive genes in Genesis836 and 7 unique salt-responsive genes in Rupali (**Table 2**), while 40 genes had genotype-dependent expression (**Table 3**) in this functional category. Upregulated DEGs due to salinity in Rupali included an early-responsive to dehydration protein (*ERD*—Ca07247) and delta-1-pyrroline-5-carboxylate dehydrogenase (*P5CDH*—Ca18937); whereas the five downregulated salt-responsive genes in Rupali, mostly involved in proline biosynthesis, were proline synthetase (Ca23258), proline dehydrogenase (Ca26838 and Ca26841), arginase family protein (*ARGAH1*—Ca04683), or raffinose synthase family protein (*SIP1*—Ca30779). Among the genes with ↑Rupali expression, nine were late embryogenesis abundant (LEA) proteins (Ca25782, Ca05155, Ca05154, Ca27912, Ca11326, Ca04033, Ca27389, Ca24842, and Ca24842) and two genes encoded dehydration-responsive RD22-like protein (Ca20394 and Ca13645). Other genes in this category included myo-inositol 1-phosphate synthase (*MIPS1*—Ca21540; *MIPS2*—Ca03667), trehalose-6-phosphate synthase (Ca16207), glutamine synthetase 1;4 (*GLN1;4*—Ca06701), polyamine oxidase 2 (*PAO2*—Ca23618), proline dehydrogenase (Ca26841), and the early-responsive to dehydration stress (ERD) family protein (Ca14703 and Ca03022).

Fifteen upregulated salt-responsive DEGs in Genesis836 involved genes encoding LEA proteins (Ca03142, Ca11285, Ca17337, and Ca23033), polyamine oxidase 1 (*PAO1*—Ca24138), dehydration-responsive protein (Ca00264 and Ca20393), *ERD4* (Ca03022), *ERD15* (Ca23212), *MIPS2* (Ca03667), spermidine synthase 3 (*SPDS3*—Ca21682), proline extensin-like receptor kinase 1 (*PERK1*—Ca01417), raffinose synthase family protein (*SIP1*—Ca19250), delta-1-pyrroline-5-carboxylate synthetase 2 (Ca27897), and glutamine synthetase 1;4 (*GLN1;4*—Ca06701). These DEGs present a better response to the osmotic component of salt stress for Genes836 than Rupali. On the other hand, downregulated DEGs in Genesis836 encoded glutamine amidotransferase-like superfamily protein (Ca10462), trehalose-phosphate phosphatase (Ca04970), and S-adenosyl-L-methionine-dependent methyltransferases protein (*ACL5*—Ca14629). Genes with ↑Genesis836 expression were associated with proline, polyamine, trehalose, and glutamine homeostasis, including polyamine oxidase (*PAO1*—Ca24138; *PAO4*—Ca06737), proline transporter (*PROT1*—Ca29910 and Ca03067; *PROT2*—Ca19219), delta1-pyrroline-5-carboxylate (*P5CS1*—Ca06790), and pyrroline-5-carboxylate reductase (*P5CR*—Ca26362). Similarly, class I glutamine amidotransferase (Ca26119 and Ca20124), GMP synthase (glutamine-hydrolysing Ca03449), glutamine-tRNA ligase (Ca12135), and trehalose-6-phosphate synthase (Ca10386 and Ca12117) exhibited ↑Genesis836 expression.

#### Salt stress signalling and regulatory pathways

Among the unique Rupali salt-responsive DEGs, one upregulated gene encoding for calcium-dependent protein kinase (*CPK13*—Ca24711) was involved in intracellular signal transduction. The remaining seven genes in this category were downregulated in Rupali under salt stress (**Table 2**) and annotated as ABA-responsive element (*AREB3*—Ca30107), calcineurin B-like protein involved in calcium-mediated signalling (*CBL2*—Ca31905) and *CBL10* (Ca16701), CBL-interacting protein kinase (*CIPK4*—Ca13161), abscisic acid-activated signalling pathway gene (*CEN1*—Ca00545), calmodulin-domain kinase CDPK protein (*CDPK2*—Ca03519), and serine/threonine-kinase SAPK1-like protein (*SNRK2.4*—Ca03741). For genotype-dependent expression, 17 DEGs with ↑Rupali expression were involved in signalling and regulatory pathways (**Table 3**), including five CDPKs [*CPK6* (Ca30481), *CRK1* (Ca26329), *CPK30* (Ca18949), *CPK8* (Ca26415), and *CRK* (Ca02486)], four CIPKs [*CIPK3* (Ca30122), *CIPK12* (Ca07182), *CIPK25* (Ca07803), and *CIPK9* (Ca17277)], and two mitogen-activated protein kinase (*MAPK3*—Ca03776; *MAPKKK5*—Ca20975).

Genesis836 had 10 unique salt-responsive genes in the salt stress signalling and regulatory pathways category (**Table 2**); the upregulated DEGs encoded ABA-induced PP2C gene 2 (*HAI2*—Ca06774), abscisic acid-responsive elements-binding factor 2 (*ABF2/AREB1*—Ca20945 and Ca02503), SNF1-related protein kinase 2.1 (*SNRK2.1*—Ca06872), and regulatory components of ABA receptor 3 (*RCAR3/PYL8*—Ca13545), and the downregulated DEGs encoded CBL-interacting protein kinase 9 (*CIPK9*—Ca17277 similar to *SOS2* with ↑Rupali expression), zeaxanthin epoxidase (*ZEP*—Ca15142), mitogen-activated protein kinase 20 (*MPK20*—Ca07390), abscisic acid-responsive family protein (*TB2/DP1/HVA22*—Ca14908), and abscisic acid receptor PYR1-like protein (*PYR1/RCAR12*—Ca01048). Genes with ↑Genesis836 expression (**Table 3**) included MAP kinase (*MAPK6*—Ca02448), CDPK-related kinase (*CRK*—Ca00557), CBL-interacting kinase (*SOS2*—Ca18734), and a calcium-dependent protein kinase (*CPK13*—Ca24711).

Genes involved in abscisic acid biosynthesis had ↑Rupali expression including abscisic acid-responsive proteins (Ca02503 and Ca14908), abscisic acid receptor PYR1-like protein (Ca01048), and phosphatidic acid phosphatase (*PAP2*—Ca05580; *SPP1*—Ca27310); however, abscisic acid-responsive protein (*TB2/DP1*—Ca14908), abscisic acid receptor PYL9-like protein (*PYL9*—Ca13545), and abscisic acid-responsive factor (*ABF2*—Ca02503) exhibited ↑Genesis836 expression.

#### Cell redox homeostasis

Plants under salt stress also experience drastically elevated levels of reactive oxygen species (ROS), which are the toxic by-products of stress metabolism and important signalling molecules. Rupali and Genesis836 each had 9 unique salt-responsive DEGs involved in cell redox (**Table 2**). Rupali had three upregulated DEGs due to salinity [encoding glutathione S-transferase family protein (Ca01231 and Ca26704) and nucleobase-ascorbate transporter (Ca15932)] and six downregulated genes were glutathione S-transferase (Ca15459, Ca31913, and Ca17240), Fe superoxide dismutase 3 (*FSD3*—Ca04671), glutathione dehydrogenase (Ca10585), and glutaredoxin family protein (*GRX*—Ca07284). Other redox genes exhibiting genotype-dependent ↑Rupali expression included two glutathione S-transferase genes (Ca15459 and Ca06563) and one microsomal glutathione S-transferase (Ca31963). Additionally, ascorbate peroxidase 3 (*APX3*—Ca03004), transmembrane ascorbate ferrireductase (Ca11792), Fe superoxide dismutase 2 (*FSD2*—Ca15694), and glutathione peroxidase (*GPX8*—Ca03496; *GPX5*—Ca10743) genes were highly expressed in Rupali control and treated samples (**Table 3**).

The Genesis836 response to salt stress resulted in six upregulated DEGs [glutathione S-transferase family protein (*GSTL3*—Ca12445 and Ca20449), glutathione S-transferase THETA 1 (*GSTT1*—Ca03490), glutaredoxin family protein (*GRX*—Ca21118 and Ca05284), and *APX3* (Ca03004—↑Rupali expression)] and three downregulated DEGs [glutathione S-transferase (*GSTU7*—Ca06563 ↑Rupali expression), glutathione S-transferase family protein (*GST30*—Ca31453), and glutaredoxin family protein (*GRX*—Ca24778)]. In addition, five genes with ↑Genesis836 expression related to cell redox homeostasis including four encoding glutathione S-transferase (*GSTU*—Ca14356, Ca05730, Ca01231, and Ca18438) and one glutathione reductase (*GR*—Ca10675).

Thus, the unique salt-responsive DEGs in Rupali and Genesis836 and DEGs exhibiting genotype-dependent expression changes indicate that the two chickpea genotypes exhibit different tolerance mechanisms to combat salt stress.

### Genotype-dependent salt-responsive DEGs (interaction)

Genes with genotype-dependent expression change due to salt treatment are of particular interest since they directly represent the difference in salt tolerance mechanisms between genotypes. Genesis836 and Rupali had 122 genes that responded to salt stress (interaction effect in the linear model). Of these, the expression of 88 genes changed direction between control and treated samples in both genotypes. The remaining 34 maintained the same direction but had manifold different expressions between genotypes. Of the 88 genes, 66 were upregulated in Genesis836 but downregulated in Rupali and 22 were downregulated in Genesis836 and upregulated in Rupali due to salinity. **Supplementary Table S7** lists all the genes in this category.

**Figure 6:**
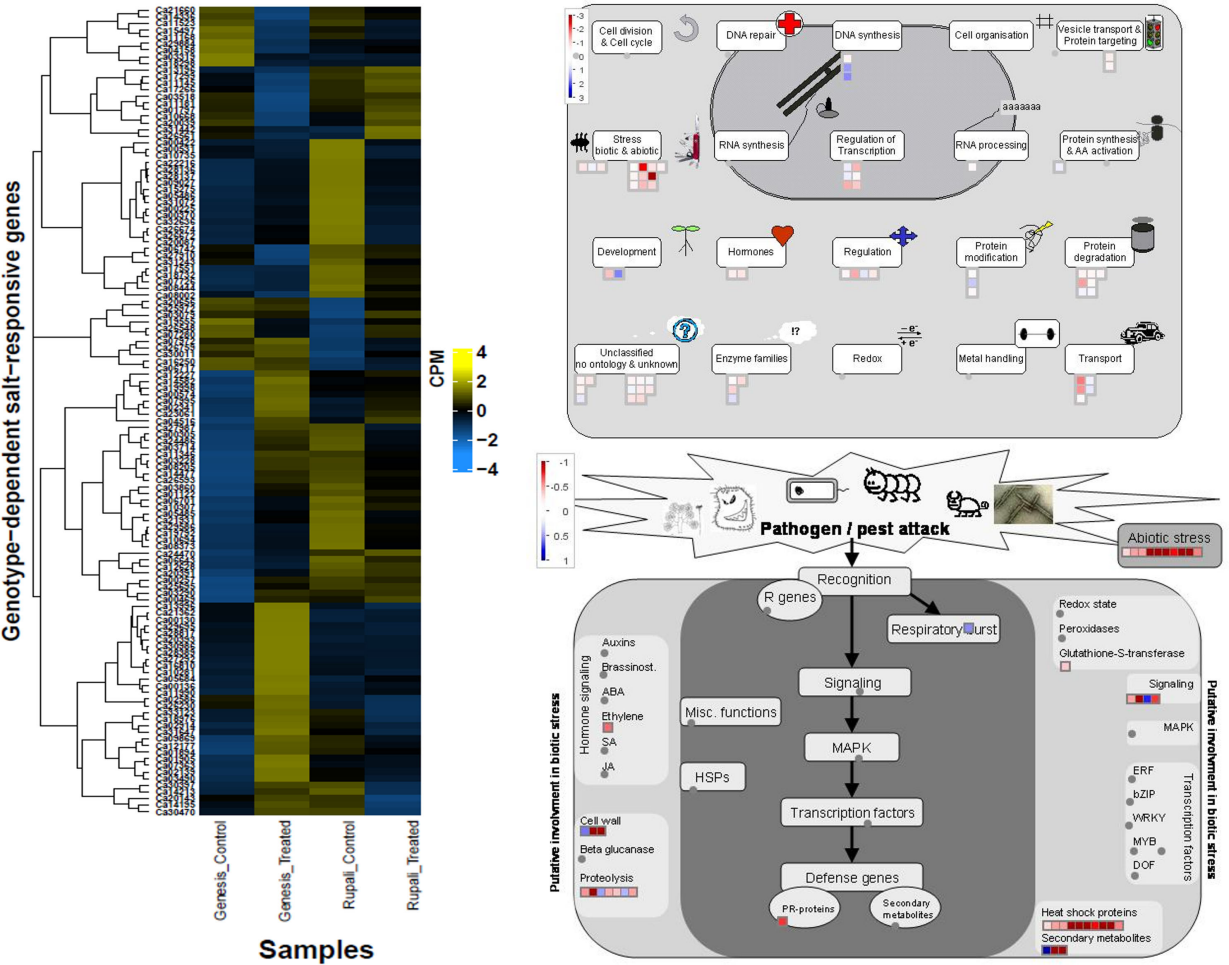
Heatmap of genotype-dependent salt-responsive DEGs and MapMan pathway views delineating the possible role of these genes.

Genes with increased expression in Genesis836 and decreased expression in Rupali included chaperone dnaJ 3-like protein (Ca08373), glutathione S-transferase (Ca03490), numerous heat shock proteins (Ca32636, Ca03714, Ca28137, Ca28136, Ca19792, Ca26672, Ca26674, Ca30397, Ca05486, Ca05485, and Ca11346), transcription factors like heat shock factors (Ca10694 and Ca00257), *bHLH* (Ca03228), *BEL1* (Ca24486), zinc fingers (Ca01122 and Ca07280), *GTE7* (Ca07363), and some stress-inducible proteins. Some genes promoted plant growth and development, such as those involved in cell wall loosening and biogenesis (Ca02914, Ca18732, Ca29695, Ca06643, and Ca14213), rate of leaf initiation (Ca27292), response to light (Ca14477, Ca12628, and Ca00370), and embryo development (Ca15275). Mapman identified these genes as mainly involved in stress, transport, regulation, and protein degradation. Genes with increased expression in Rupali and decreased expression in Genesis836 include glucose-1-phosphate adenylyltransferase (Ca29884), calcium transporting ATPase (*ACA11*—Ca13196; *ECA2*—Ca17299), auxin response factor (*ARF*—Ca03319), brassinosteroid signalling pathway protein (*BSK3*—Ca04158), respiratory burst oxidase homolog protein (Ca03518), TFs such as zinc finger (Ca11181 and Ca07280) and winged-helix DNA-binding factor (Ca10668 and Ca26561), genes involved in cell wall biogenesis (Ca25972 and Ca11145), and proteins involved in cell death (Ca13196 and Ca03518). These 22 genes were mainly involved in DNA synthesis, protein modification, and development. Mapman identified some interesting groups of genes encoding heat shock proteins/factors, transport, and regulatory proteins in this DEGs category (**Figure 6**), which were induced in Genesis836 but repressed in Rupali.

Thus, the study highlights many potential candidate genes and their roles in salt response and tolerance mechanisms in the two chickpea genotypes, which can be further potential candidates for breeding salt-tolerant chickpea cultivars.

### Validation of differentially expressed genes

**Figure 7:**
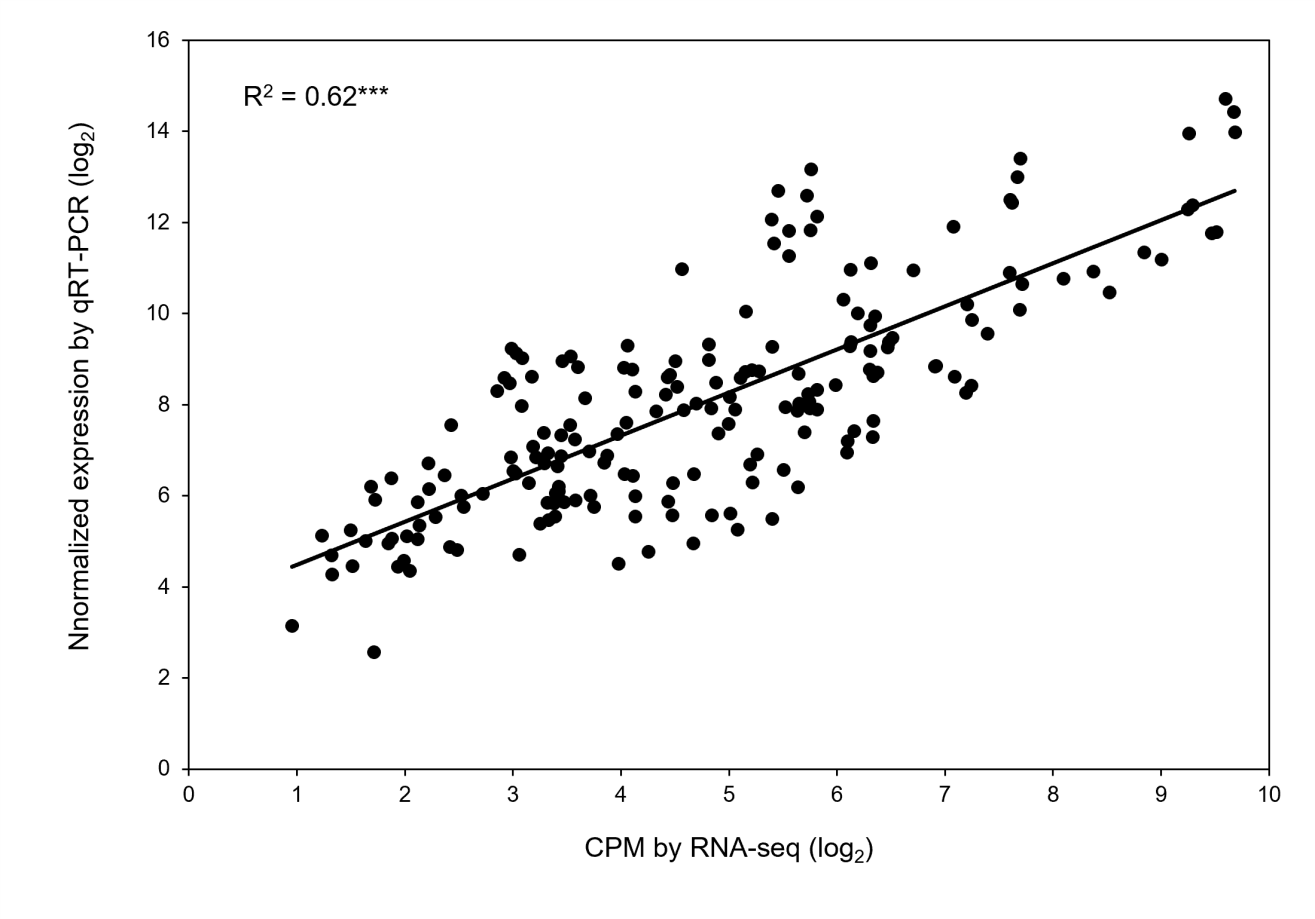
Validation of differential expression of genes obtained by RNA-seq. Correlation of gene expression results obtained from RNA-seq and quantitative real-time PCR for eight randomly selected genes. Each value is an individual replication from different treatment combinations. *** Significant with *p*-value < 0.001.

Quantitative real-time PCR (qRT-PCR) was performed on RNA samples for eight genes (Ca30477, Ca13456, Ca02100, Ca14863, Ca10383, Ca29966, Ca19227, and Ca01215) randomly selected to validate the RNA-seq results. **Table 4** lists the gene-specific primer sequences. A significant correlation occurred between the relative expression levels obtained from RNA-seq and qRT-PCR analysis (*r*^2^ = 0.62 with *P* < 0.001; **Figure 7**), validating the biological significance of RNA-seq data.

**Table 4:** List of primer sequences used for quantitative real-time PCR (qRT-PCR) analysis.

Gene ID	Product length	Primer
Ca30477	189	F - 5′-GGTCCGCTTCTTGTGGTC-3′
		R - 5′-AATCCTCCTGTGGCTTGATG-3′
Ca13456	184	F - 5′-CTGAAGAGAGTAGTTGTGTTTGG-3′
		R - 5′-GACTTTGTTGATTGTTGTTGAATG-3′
Ca02100	103	F - 5′-CCAACCTCGTCGGAACTATC-3′
		R - 5′-AGCGTGGTGGGTATCTCG-3′
Ca14863	198	F - 5′-CAACTCTGTCTCATCATCATCATC-3′
		R - 5′-TGCCAAGTCCAAGTCCAAC-3′
Ca10383	196	F - 5′-TCCACCAGCCAAAGTCTC-3′
		R - 5′-GTCCTCCATTTCCTCTAAACC-3′
Ca29966	187	F - 5′-GGAACTCGCACTGTCATTG-3′
		R - 5′-GTGTCCTTTGCCAACTCTTC-3′
Ca19227	178	F - 5′-TGGGCAATACAAAGAGACC-3′
		R - 5′-CTAACACATATTCAACAAGAGC-3′
Ca01215	193	F - 5′-GCTGCTGTTCAAAGATATAGAG-3′
		R - 5′-GCATAGTTCTTGGATATTCACC-3′

### Variants in the two genotypes

Uniquely mapped reads were used to identify variants in Rupali and Genesis836. We identified 13,829 variants in 6,484 genes in Genesis839 and 14,002 variants in 6,564 genes in Rupali compared to the reference genome. Of these, 1,449 variants were present in 701 Rupali salt-responsive DEGs and 1,741 variants were present in 768 Genesis836 salt-responsive DEGs (**Figure 4**; **Supplementary Table S8**).

Furthermore, stop_gained mutations were particularly interesting as they indicate premature stop codons and knockouts. Forty variants in 35 genes of the salt-sensitive variety Rupali were stop_gained mutations (**Supplementary Table S9**). In other words, 35 genes were knocked out due to premature stop codons, suggesting that salt intolerance in the sensitive genotype Rupali is a consequence of loss of function. Of the 35 genes, Ca20374 and Ca17320 were related to salt stress tolerance: Ca20374 is a predicted B-box zinc finger protein, which encodes a transcription factor BBX20 (also named STH7/salt tolerance homolog 7), involved in the brassinosteroid-mediated signalling pathway and photomorphogenesis and Ca17320 is a phosphoinositide kinase involved in protein autophosphorylation and response to salt stress.

Other DEGs with stop codons included a peptidyl-prolyl cis-trans isomerase FKBP62-like protein (Ca16597) involved in response to heat and osmotic stress, a phosphoenolpyruvate carboxylase kinase 1 (*PPCK1* Ca10979) whose expression is induced by light and involved in signal transduction and phosphorylation, an RNA polymerase II transcription subunit (Ca06996) involved in regulating hormone-mediated signalling pathways and photoperiodism, and a gene involved in morphogenesis, growth, and meristem development (Ca03638) (**Supplementary Table S9**). Moreover, Ca16824, Ca02694, and Ca16823 are involved in vesicle transport. TF genes with knockout mutations included Ca02188 (RING-H2 finger protein), Ca07014 (AP2/B3 transcription factor family protein), Ca10947 (zinc finger CCCH domain-containing protein), and Ca15012 (auxin response factor protein). Ten genes containing stop_gained mutations were uncharacterised and may serve as potential candidates for further evaluation (**Supplementary Table S9**).

### DEGs associated with salinity tolerance QTLs

A recently published study developed a Recombinant Inbred Line (RIL) population from a cross between Rupali (salt-sensitive) and Genesis836 (salt-tolerant) chickpea (Atieno et al., 2021) and reported three specific salinity tolerance QTLs, i.e., Ca4 (6.8–7.5 Mb), Ca5 (10.4-21.4 Mb), and Ca6 (13.8-15.0 Mb) from the coordinates of flanking markers based on CDC Frontier v.2.6.3. Thus, we investigated our DEGs categories for genes positioned within the three QTL regions (**Supplementary Tables S2**, **S3**, **S5**, **S7**). Among the DEGs positioned within these QTLs, 15 genes were identified with a potential role in chickpea salt tolerance (**Table 5**), which included Ca20374 at Ca5 with a stop_gained mutation in Rupali, a thylakoid lumenal 17.9-kDa protein (Ca21063 at Ca5) and a glutathione S-transferase-related protein (Ca20449 Ca5). Moreover, some dehydration-responsive proteins, heat shock proteins, and MYB-like transcription factor family proteins were among the DEGs within the salinity tolerance QTLs.

**Table 5:** DEGs located within the QTLs associated with salinity tolerance.

Gene ID	Chr	Start	End	logFC	Chickpea Annotation
Rupali salt-responsive DEGs					
**Ca11286**	Ca4	7138881	7145407	2.358244	MYB transcription factor
Genesis836 salt-responsive DEGs					
Ca11285	Ca4	7105683	7108605	0.477233	Late embryogenesis abundant protein (LEA) family protein
**Ca11286**	Ca4	7138881	7145407	1.480611	MYB transcription factor
Ca20374	Ca5	13265759	13267511	−1.08576	B-box zinc finger protein 20-like, salt tolerance-like protein
**Ca20393**	Ca5	13521975	13523006	1.221774	BURP domain-containing protein 3-like, dehydration-responsive RD22-like protein
Ca20449	Ca5	14332277	14334948	1.219026	Prostaglandin E synthase 2-like, Glutathione S-transferase family protein
**Ca20868**	Ca5	14977203	14979976	−1.00635	Putative uncharacterized protein, MYB family transcription factor
**Ca20497**	Ca5	15082134	15087579	−0.68143	J domain-containing protein required for chloroplast accumulation response 1 isoform X1, heat Shock protein-binding protein
**Ca03290**	Ca6	14180788	14182071	1.986351	Uncharacterized protein, MYB-like transcription factor family protein
Ca03319	Ca6	14311213	14318255	−0.60831	Auxin response factor 19-like isoform X1
DEGs with Genotype-dependent expression differences					
**Ca11286**	Ca4	7138881	7145407	−3.10623	MYB transcription factor
Ca11326	Ca4	7445931	7447103	1.708474	Indole-3-acetic acid-induced protein ARG2-like, late embryogenesis abundant protein
Ca20373	Ca5	13279009	13280172	1.158472	Dehydrin ERD14-like
**Ca20393**	Ca5	13521975	13523006	−1.94947	BURP domain-containing protein 3-like, dehydration-responsive RD22-like protein
Ca20394	Ca5	13535998	13537198	2.64486	Dehydration-responsive protein RD22-like
**Ca20868**	Ca5	14977203	14979976	1.741461	Putative uncharacterized protein, MYB family transcription factor
**Ca20497**	Ca5	15082134	15087579	2.304589	J domain-containing protein required for chloroplast accumulation response 1 isoform X1, heat Shock protein-binding protein
Ca20824	Ca5	18279519	18281518	−1.35978	Heat shock factor protein HSF30
Ca21001	Ca5	19764219	19771126	−1.19929	Uncharacterized protein, myb-like DNA-binding domain protein
Ca21063	Ca5	20304677	20307502	0.724988	Thylakoid lumenal 17.9 kDa protein - chloroplastic
**Ca03290**	Ca6	14180788	14182071	2.055389	Uncharacterized protein, MYB-like transcription factor family protein
Genotype-dependent salt-responsive DEGs					
**Ca03290**	Ca6	14180788	14182071	−1.97178	Uncharacterized protein, MYB-like transcription factor family protein
Ca03319	Ca6	14311213	14318255	0.642919	Auxin response factor 19-like isoform X1

The gene names highlighted in bold letters are common between different comparisons.

## Discussion

Several studies have reported that salt stress induces complex regulatory mechanisms and major transcriptional reorganisation in chickpeas. However, the molecular mechanisms for salt tolerance and the different adaptive responses in salt-tolerant Genesis836 and salt-sensitive Rupali have not been explored. Some studies on Genesis836 and Rupali have demonstrated that Na^+^ exclusion mainly determines salt sensitivity (Khan et al., 2016) and the tissue tolerance to Na^+^ differed between the two genotypes (Khan et al., 2017), with Genesis836 moving ions from photosynthetically active mesophyll tissues into epidermal cells (Kotula et al., 2019). Hence, identifying DEGs in Genesis836 and Rupali can shed light on their tolerance mechanisms and determine what contributes to the enhanced salinity tolerance in Genesis836.

This study compared the transcriptome profiles of Genesis836 and Rupali under salt stress. Both genotypes had a similar number of salt-responsive DEGs, indicating that salinity highly disturbed these genotypes at the transcriptional level. Detailed investigations of Genesis836 and Rupali gene expression profiles grouped the DEGs as salt-responsive, genotype-dependent salt-responsive, and genotype-dependent and treatment-dependent, providing novel insights into the molecular processes involved in salt tolerance. Comparing the control vs. treated samples revealed unique salt-responsive genes in both genotypes. Such unique genes have also been identified in other susceptible and tolerant chickpea genotypes under salt stress (Kumar et al., 2021). The unique salt-responsive genes in each genotype, genotype-dependent expression differences, and genotype-dependent salt-responsive genes collectively indicate that tolerant Genesis836 and sensitive Rupali have distinct salinity responses and tolerance mechanisms. Our study agrees with previous physiological results (Kotula et al., 2019) that tissue tolerance *via* leaf Na^+^ regulation and photosynthetic maintenance are the key mechanisms that differ in Genesis836 and Rupali.

Genesis836 maintained higher shoot and root growth than Rupali with similar leaf Na^+^ concentrations, consistent with our previous findings (Khan et al., 2016; Khan et al., 2017) and suggests higher tissue tolerance to leaf Na^+^ ion in Genesis836. Plants maintain adequate growth by keeping low Na^+^ levels in the cytoplasm (Munns and Tester, 2008) or storing Na^+^ away from photosynthetic tissues. Principally, this is achieved by excluding Na^+^ from the cell cytosol *via* plasma membrane Na^+^/H^+^ antiporters (SOS1) or sequestration of Na^+^/K^+^ into vacuoles *via* tonoplast Na^+^/H^+^ antiporters (NHXs) while using energy from H^+^-ATPase in the plasma membrane or V-ATPase (VHA and AVA) and H^+^-PPase (VP) in intracellular compartments (Bassil and Blumwald, 2014). Our results delineated the upregulation of a tonoplast-localised Na^+^/H^+^ antiporter (*NHX2*) under salt-stressed Genesis836 in combination with Na^+^/H^+^ antiporter (*NHD1*) and genes encoding vacuolar proton ATPase and assembly proteins (*VHA-A2*, *VHA-B-like* and *VMA21-like*), all exhibiting ↑Genesis836 expression, suggesting improved Na^+^ sequestration into vacuoles. Nevertheless, Rupali uniquely upregulated vacuolar H^+^-translocating inorganic pyrophosphatase (*VP1*), along with two genes encoding plasma membrane H^+^-ATPase (*HA1*) with ↑Rupali expression. Coordination between Na^+^ antiporters and vacuolar H^+^-ATPase and H^+^-PPase activities is critical for Na^+^ compartmentalisation in vacuoles (Hasegawa, 2013). In addition, the electrochemical gradient of H^+^ generated by vacuolar H^+^-ATPase and H^+^-PPase might have provided additional energy for *NHX* activity to transport Na^+^ against high vacuolar concentrations in Genesis836 (Peleg et al., 2011).

High-affinity K^+^ transporter proteins (HKTs) also reduce shoot Na^+^ by removing Na^+^ from the xylem and keeping it to roots (Davenport et al., 2007). However, in *Arabidopsis* leaves, AtHKT1 is expressed in the plasma membrane in xylem parenchyma cells, selectively unloading Na^+^ directly from xylem vessels to xylem parenchyma cells and protecting plant leaves from salinity stress (Sunarpi et al., 2005). Moreover, time- and tissue-dependent expression of *AtHKT1* determines Na^+^ distribution in plant organs/tissues (Hamamoto et al., 2015). We observed downregulation of *HKT1* in Rupali under stress, but the gene showed ↑Rupali expression compared to Genesis836. Similarly, a tolerant rice genotype had lower *HKT1* expression in shoots and roots than a sensitive genotype (Kader and Lindberg, 2005; Kader et al., 2006; Zhou et al., 2016). In wheat, the *Nax1* and *Nax2* loci (HKTs) prevented higher Na^+^ concentrations in leaf blades by unloading more Na^+^ from the xylem into the leaf sheath (James et al., 2006; James et al., 2011). Higher expression of *HKT1* in Rupali tissues indicates a higher Na^+^ influx into the cytosol of mesophyll cells, making it more salt-sensitive. Similarly, *HKT1* expression may also differ between chickpea tissue types, e.g., petioles and leaflets, affecting the unloading and storage of more Na^+^ into petioles and avoiding high Na^+^ concentrations in leaf blades. Therefore, investigating the cell-specific expression of *HKT1* will help understand how its expression influences Na^+^ distribution in Genesis836 and Rupali leaf tissues.

Salt overly sensitive (SOS) is another important signalling pathway regulating Na^+^ efflux from root cytosol (Ji et al., 2013). The pathway comprises a Ca^2+^-binding protein *SOS3* (calcineurin B-like protein—CBL), interacting with *SOS2* (*CIPK24*) to form a protein kinase complex that upregulates the expression of *SOS1*, a putative plasma membrane Na^+^/H^+^ antiporter responsible for Na^+^ efflux from the cytoplasm (Shi et al., 2002; Ji et al., 2013). Moreover, *SOS1* also requires an H^+^ gradient generated by the plasma membrane H^+^-ATPase. Although our study found increased expression of *SOS3*, *SOS3-like* (*CIPK10* and *CIPK11*), and H^+^-ATPase (*HA1*) in Rupali leaves and *SOS2/CIPK24* in Genesis836, *SOS1* was not detected as differentially expressed. SOS2/CIPK24 forms a protein complex with CBL10 and then interacts with *NHX*s to regulate Na^+^ transport across the tonoplast in shoots (Qiu et al., 2004; Kim et al., 2007; Quan et al., 2007; Luan et al., 2009). Both *SOS2/CIPK24* and *CBL10* had ↑Genesis836 expression, which suggests facilitated *NHX* activity to sequestrate more Na^+^ into the vacuoles of Genesis836 leaves.

**Figure 8:**
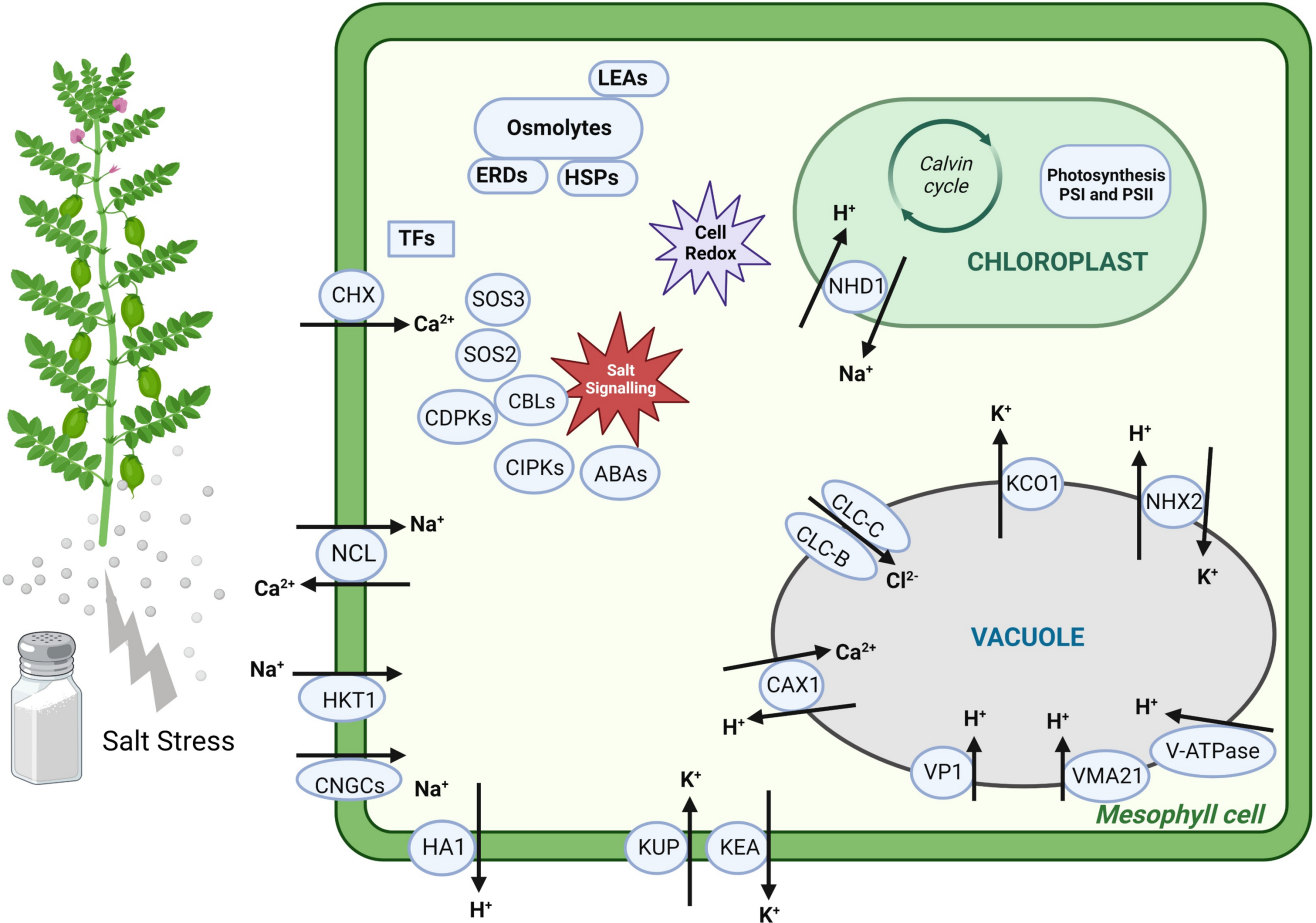
The schematic representation of some potential candidate genes involved in various biological mechanisms and differentially expressed between the two genotypes.

Most DEGs related to Cl^−^ transport exhibited ↑Genesis836 expression, such as genes encoding Cl^−^ conductance regulatory protein ICln protein and Cl^−^ channel proteins (CLC-B and CLC-C), whereas only one gene encoding Cl^−^ channel (CLC-C) delineated ↑Rupali expression. CLC-C is a vacuolar Cl^−^ transporter linked to salt tolerance in several studies (Jossier et al., 2010; Wei et al., 2013; Henderson et al., 2014). Hence, upregulation under salinity and higher expression of *CLC-C* in Genesis836 might have contributed to enhanced tissue tolerance to Cl^−^. These DEGs indicate that the ion transport mechanisms differ between the two genotypes and appear to function better in the tolerant chickpea genotype. All the potential candidate genes involved in ion transport with known physiological and/or molecular regulation are described in **Figure 8**.

Salt stress also reduced photosynthesis in salt-sensitive Rupali through photosystem II damage (Khan et al., 2016; Khan et al., 2017) due to structural damage in the chloroplasts from increased Na^+^ accumulation in mesophyll cells (Kotula et al., 2019). Rupali under salt stress downregulated several DEGs involving the photosystem II reaction centre, thylakoid lumen, and plastid proteins, while genes encoding light-harvesting complex I chlorophyll A/B-binding proteins exhibited ↑Rupali expression. In contrast, salt-stressed Genesis836 downregulated most of the light-harvesting chlorophyll A/B-binding proteins and photosystem I-associated and chloroplast-associated proteins, and upregulated genes related to plastid development. In addition, several genes exhibiting ↑Genesis836 expression, mainly related to plastid development, RuBisCo binding and function, and chlorophyll-binding proteins, may contribute to salt tolerance in Genesis836. Various studies have reported the effect of salt stress on photosynthesis and photosystem damage in diverse crops (reviewed by Zahra et al., 2022); however, the combination of physiological and molecular insights remains limited and our study assessed both aspects of salt tolerance in two chickpea genotypes exhibiting contrasting phenotypes.

Ascorbate–glutathione is a crucial ROS-scavenging pathway that responds to oxidative stress and correlates with improved photosynthesis and salt tolerance in plants (Tang et al., 2015). The main components of the ascorbate–glutathione pathway are genes encoding glutathione S-transferase (GST), glutathione reductase (GR), glutathione peroxidase (GPX), and ascorbate peroxidase 3 (APX3). DEGs encoding GST were upregulated in Genesis836 under salt stress. DEGs encoding GR and glutaredoxin family proteins (GRX) were highly expressed in Genesis836 tissues, whereas Fe superoxide dismutase 2 (*FSD2*) and *GPX5* and *GPX8* had ↑Rupali expression. GR mainly operates in photosynthetic tissues (Gill et al., 2013), playing an essential role in the glutathione redox state to protect rice plants against salt stress (Wu et al., 2015). Thus, these results support that Genesis836 improves photosynthesis *via* maintaining better functional photosystem against ion toxicity and ROS damage than Rupali.

Our analysis also identified numerous DEGs encoding osmoprotectants, such as genes related to proline/pyrroline metabolism and transport (*P5CS1, P5CR, PERK1, PROT1*, and *PROT2*), polyamine oxidase (*PAO1* and *PAO4*), myo-inositol phosphates hydrolysis (myo-inositol polyphosphate 5-phosphatase), biosynthesis of raffinose (*SIP1*), spermine (*SPDS3*), glutamine (*GLN1;4*), trehalose, and dehydration response proteins, which were upregulated under salt stress or highly expressed in Genesis836. In contrast, numerous genes encoding LEA proteins, *MIPS1*, and *MIPS2* delineated ↑Rupali expression. Besides osmoregulation, proline protects cell membranes and scavenges radicals under salt stress; hence, proline transport and accumulation are important components of adaptive mechanisms for tolerating salinity (Ueda et al., 2001). Thus, our results are indicative of osmoprotectants promoting salt tolerance in Genesis836. Increased production of myo-inositol and trehalose contributes to salt tolerance in many species (Jang et al., 2003; Avonce et al., 2004; Majee et al., 2004; Das-Chatterjee et al., 2006; Perera et al., 2008; Kaye et al., 2011; Li et al., 2011). Our RNA-seq study highlighted several osmoprotectant genes as possible candidates for osmotic tolerance in the two chickpea genotypes.

Our results primarily presented three groups of protein kinases among the DEG list: calcium-dependent protein kinase (CDPK/CPK), CBL-interacting protein kinase (CIPK), and mitogen-activated protein kinase (MAPK). In our study, most of the DEGs corresponding to CIPKs and CPKs (*CPK6, CPK30, CPK8, CIPK3, CIPK12, CIPK25, CPK33*, and *CIPK9*) exhibited ↑Rupali expression. *CPK30* is a positive regulator of stress response in barley (Sheen, 1996). CDPKs are generally positive regulators of abiotic stress response and enhanced expression of CDPKs increased salt tolerance (Boudsocq and Sheen, 2013; Thoday-Kennedy et al., 2015). *MAPK6* exhibited ↑Genesis836 expression and its over-expression correlated with increased salt tolerance in *Arabidopsis* (Ichimura et al., 2000). Lower expression of *MAPK3* and *MAPKKK5* in Genesis836 is supported by earlier findings where the activation of *MAPK3* mediated salt sensitivity in rice *via* signalling interactions and ultimately increased plant survival (Li et al., 2014). Overall, the protein kinases identified in our study were either downregulated under salinity or had comparatively lower expression in Genesis836 than Rupali, except *CPK13* and *MAPK6*, indicating a possible role of protein kinases in genotypic variation under salt stress.

In addition, the genotype-dependent salt-responsive genes represent another category of DEGs that responded differently to salt stress in Genesis836 and Rupali. These DEGs mainly comprised heat shock, transport, and regulatory proteins, which were induced in Genesis836 and repressed in Rupali. DEGs within the salinity tolerance QTLs were involved in various processes for salt tolerance, e.g., photosystem activity, antioxidative pathway, and stress response such as MYB TFs, dehydration-responsive proteins, and heat shock proteins. Furthermore, the salt-sensitive Rupali contained many stop_gained mutations, including two genes (Ca20374 and Ca17320) with a known role in salt stress tolerance, which will be useful for future investigations.

Overall, we found that genotypic variability in salt tolerance is attributed mainly to differences in the expression of genes involved in Na^+^ transport and photosynthetic maintenance. Moreover, we identified uncharacterised DEGs without known functions in abiotic stress tolerance, serving as ideal candidates for further experimental validations. Hence, this study highlighted the role of potential candidate DEGs and their regulatory networks in salt tolerance, as described in **Figure 8** based on their biological role, which can be used to breed salt-tolerant chickpea cultivars.

## Conclusion

This study presents a comprehensive analysis of the leaf transcriptome of two chickpea genotypes contrasting in response to salt stress. The comparative differential expression of genes between salt-sensitive Rupali and salt-tolerant Genesis836, together with previously identified physiological mechanisms, extends our understanding of the cultivar-specific molecular mechanisms contributing to salt tolerance in chickpeas. Most of the DEGs identified in the tolerant and susceptible phenotypes were related to sodium transport, photosynthesis, stress signalling, cell redox homeostasis, and heat shock proteins, which serve as a valuable repository of candidate genes for developing salt-tolerant chickpea varieties.

## Data availability statement

The datasets presented in this study can be found in online repositories. The names of the repository/repositories and accession number(s) can be found in the article/**Supplementary Material.**

## Author contributions

HK performed experiments, prepared tables and figures, and wrote the first draft of the manuscript. NS carried out bioinformatics analysis, prepared tables and figures, and wrote the first draft of the manuscript. UB supervised bioinformatics analysis, KS and TC supervised the project, and TS supervised the study and project. All authors read and contributed to the revised version of the manuscript.
